# COVID‐19 Mortality in Swedish Intensive Care Units: A Multicenter Survival Analysis

**DOI:** 10.1111/aas.70279

**Published:** 2026-06-14

**Authors:** Gustaf Forsberg, Knut Taxbro, Sören Berg, Fredrik Hammarskjöld, Johan Berkius, Håkan Johansson, Andreas Ekman, Åse Östholm, Katarina Niward, Jonna Idh, Louise Elander

**Affiliations:** ^1^ Department of Cardiothoracic and Vascular Surgery Linköping University Hospital Linköping Sweden; ^2^ Department of Health, Medicine and Caring Sciences Linköping University Linköping Sweden; ^3^ Department of Biomedical and Clinical Sciences Linköping University Linköping Sweden; ^4^ Department of Anaesthesiology and Intensive Care Medicine Ryhov County Hospital Jönköping Sweden; ^5^ Helicopter Emergency Medical Service, Västra Götaland Region Gothenburg Sweden; ^6^ Department of Anaesthesiology and Intensive Care Västervik Hospital Västervik Sweden; ^7^ Department of Research Region Kalmar County Kalmar Sweden; ^8^ Department of Anaesthesia Trelleborg Hospital, Skåne University Hospital Malmö Sweden; ^9^ Department of Anaesthesiology and Intensive Care Kalmar Hospital Kalmar Sweden; ^10^ Department of Clinical Sciences Lund University Lund Sweden; ^11^ Department of Infectious Diseases Linköping University Hospital Linköping Sweden; ^12^ Department of Anaesthesiology and Intensive Care Linköping University Hospital Linköping Sweden; ^13^ Department of Global Public Health Karolinska Institutet Stockholm Sweden; ^14^ Centre for Clinical Research Sörmland, Uppsala University Eskilstuna Sweden; ^15^ Department of Anaesthesiology and Intensive Care Nyköping Hospital Nyköping Sweden

**Keywords:** acute respiratory distress syndrome (ARDS), COVID‐19, intensive care, multicenter, SARS‐CoV‐2, Sweden, treatment strategies

## Abstract

**Background:**

Mortality among critically ill COVID‐19 patients has varied globally. In Sweden, geographic differences in mortality have also been observed. The current study aimed to determine whether mortality differences persist after adjusting for differences in case‐mix, and to identify potential independent factors contributing to regional variations in mortality.

**Methods:**

We conducted a multicenter cohort study including adult patients admitted to seven hospital ICUs across three Swedish healthcare counties between March 1, 2020 and July 31, 2021. These ICUs include one university hospital, three county hospitals and three local hospitals, and cover the intensive care infrastructure for approximately one million inhabitants. Patients were assigned to the hospital of initial ICU admission, even if transferred later during the course. Patient characteristics, disease severity, respiratory support, and treatments were registered. Primary outcome was 90‐day mortality. A mixed‐effects Cox proportional hazards model was used.

**Results:**

Seven hundred and forty seven patients were included. The unadjusted 90‐day mortality varied significantly, with the highest rate at 30%, and the lowest at 8.5% (*p* < 0.001). After adjustment for baseline confounders (Charlson comorbidity index, sex, SAPS3, age, smoking status, BMI), calendar time and healthcare county (random intercept), all hospitals were significantly associated with increased 90‐day mortality compared with the lowest mortality hospital. Hazard ratios ranged from 2.38 to 5.06.

**Conclusion:**

Among patients admitted to ICU due to COVID‐19, we observed a difference in mortality related to the hospital of first ICU admission. This difference persisted after adjustment for calendar time, baseline confounders, and healthcare county. Potential explanations are lacking within the current study. Future studies should focus on comprehensive evaluation of both organizational and contextual determinants of mortality.

**Editorial Comment:**

This analysis from 3 Swedish counties (7 hospitals) for COVID ICU cases presents factors and relations to mortality risk, including factors for first admission to university‐larger‐, or smaller hospital. An association was observed for higher risk if the first ICU admission was in a smaller hospital, though recognizing that this is a dataset coming from a small set of hospitals.

## 1 Background

Since its emergence in late 2019, the World Health Organization (WHO) estimates that coronavirus disease 2019 (COVID‐19) contributed to approximately 14.83 million global excess deaths during 2020–2021 [1]. Recently, Pizzato et al. published an analysis of excess mortality across Europe during the pandemic (2020–2023), estimating approximately 1.6 million excess deaths. The age‐standardized excess death rate per 10,000 inhabitants ranged from 1.8 in Sweden to 24.7 in Bulgaria [2]. These disparities in mortality between countries may be attributed to a variety of factors, including case‐mix, ethnicity [3], socioeconomic conditions [4], healthcare practices, staffing levels, and access to materials and medications.

High bed occupancy and increased patient‐to‐nurse ratio have been associated with higher mortality and morbidity [5, 6]. Surges of COVID patients during the pandemic increased both patient‐to‐nurse ratios and necessitated the deployment of staff not routinely working in intensive care, both of which were associated with increased mortality and adverse event rates [7]. Limited capacity necessitated the rapid development of admission triage guidelines to allocate and maximize overall benefit of the available resources [8]. A Swedish study identified high intensive care unit (ICU) strain to be associated with a higher degree of invasive respiratory support, suggesting that resources were used for most severe cases during capacity limits [9].

International studies of critically ill ICU‐treated COVID‐19 patients have shown large variations in outcome. In a large US study of COVID‐19 ICU patients, a wide variation of mortality was seen, ranging from 0% to 82%. This variability was related to socio‐economic factors, hospital capacity, and patient physiologic parameters [10]. In a previous Swedish study, mortality among ICU‐treated COVID‐19 patients appeared to vary between hospitals [11]. However, adjusted analyses are lacking. We hypothesized that 90‐day mortality among ICU‐treated COVID‐19 patients would vary between hospitals and that this variation would be related to patient‐level case‐mix. The aim of the present study was therefore to examine whether mortality differed according to the hospital of initial ICU admission using adjusted analyses accounting for patient‐level factors, treating the hospital of admission as a contextual exposure reflecting difference in care environments.

## 2 Methods

### 2.1 Primary Research Question

1. Does 90‐day mortality among COVID‐19 ICU patients vary by the hospital of initial ICU admission, and does this variation remain after adjustment for case‐mix?

### 2.2 Secondary Research Questions

1. What inter‐hospital differences in intensive care management of COVID‐19 patients can be identified?
2. Do these differences align with the mortality variation observed between hospitals?

#### 2.2.1 Study Design, Setting and Outcomes

We performed a population‐based multicenter retrospective cohort study including all ICUs from seven hospitals, within three healthcare counties in Sweden, covering a total population of one million inhabitants. County 1 includes three hospitals with ICUs at two of them (A1 and B1). County 2 comprises three hospitals equipped with ICUs (B2, C1, C2). County 3 includes three hospitals, two of which have ICUs (B3 and C3). Hospital A1 is a university hospital, B1‐3 are county hospitals, and C1‐3 are local hospitals. All patients 18 years of age or older admitted to an ICU with acute hypoxemic respiratory failure due to COVID‐19 from March 1, 2020 to July 31, 2021 were included. The study inclusion period was chosen to capture the initial phase of the COVID‐19 pandemic. At the time, ICU care pathways, treatment strategies and resource availability were still under development, providing an opportunity to study how early local management strategies and organizational responses potentially affected patient outcomes. The primary exposure was the hospital of first ICU admission, conceptualized as a contextual exposure reflecting the institutional, organizational, and clinical environment in which early ICU care was provided. Patients transferred to another ICU were recorded as belonging to the first ICU of admission, as patient transfer was considered as being an integral part of patient and resource handling. Consequently, transferred patients received part of their care at hospitals other than the one to which they were assigned. Timing of transfers was not available. Patient characteristics including ICU management, treatments, clinical course, and ICU complications were registered. Primary outcome was 90‐day mortality.

#### 2.2.2 Ethics Statement

The study was approved by the Ethical Review Board in Sweden (Ref no. 2020‐06015 and 2021‐01701). As a retrospective observational cohort study, the need for written consent was waived.

#### 2.2.3 Definitions

Hospital refers to the hospital and ICU of initial admission. 90‐day mortality is defined as all‐cause mortality within 90 days from ICU admission. Date of death was obtained through electronic medical journals and its linkage with the Swedish personal identity number. Acute respiratory distress syndrome (ARDS) was defined according to the Berlin definition [12], as adopted by the Swedish intensive care registry (SIR) [13] and reported as the most severe degree of ARDS developed. COVID‐19 is defined as rtPCR‐confirmed infection with severe respiratory syndrome coronavirus 2 (SARS‐CoV‐2). Corticosteroid treatment was defined as any treatment started with dexamethasone, betamethasone, prednisolone, hydrocortisone, or methylprednisolone for a duration of at least 48 h. Comorbidity was defined as per the international classification of diseases and related health problems revision 10 (ICD‐10) resulting in a Charlson comorbidity index (CCI) for participants, excluding age [14].

#### 2.2.4 Data Collection

Identification of patients was done by ICD codes of COVID‐19 infection (both primary and secondary) on ICU admitted patients in SIR. All identified patients were manually screened and only those admitted due to COVID‐19 hypoxemic respiratory failure were included. Clinical data were collected from medical records and from SIR. Variables included were age, sex, comorbidity, disease severity (Simplified Acute Physiology Score [SAPS3]), respiratory support (high‐flow nasal oxygen [HFNO], non‐invasive ventilation [NIV], invasive mechanical ventilation [IMV], tracheostomy), pharmacological treatment (corticosteroids, antibiotics, antivirals, and anticoagulation), dialysis, complications (readmission, reintubation). Transfers between ICUs were registered as a dichotomous yes/no variable, and underlying reasons for transfers were not registered. Data were collected and stored using Castor EDC [15].

#### 2.2.5 Statistics

All analyses were performed using R Statistical Software (v4.4.2; R Core Team 2024) and RStudio Team (2020). (RStudio: Integrated Development for R. RStudio, PBC, Boston, MA URL http://www.rstudio.com/). Relevant code used for the analysis are available as supplementary Quarto pdf. A *p*‐value of < 0.05 was considered significant.

#### 2.2.6 Descriptives

For all descriptive statistics, missing data were handled through listwise deletion. No analysis of missing data was performed for these analyses. Normally distributed continuous data are presented as mean (±SD), non‐normally distributed as median (IQR), and categorical data as a percentage. A Kaplan‐Meier curve was made using the survminer package in R.

#### 2.2.7 Directed Acyclic Graph and Variable Selection

**FIGURE 1:**
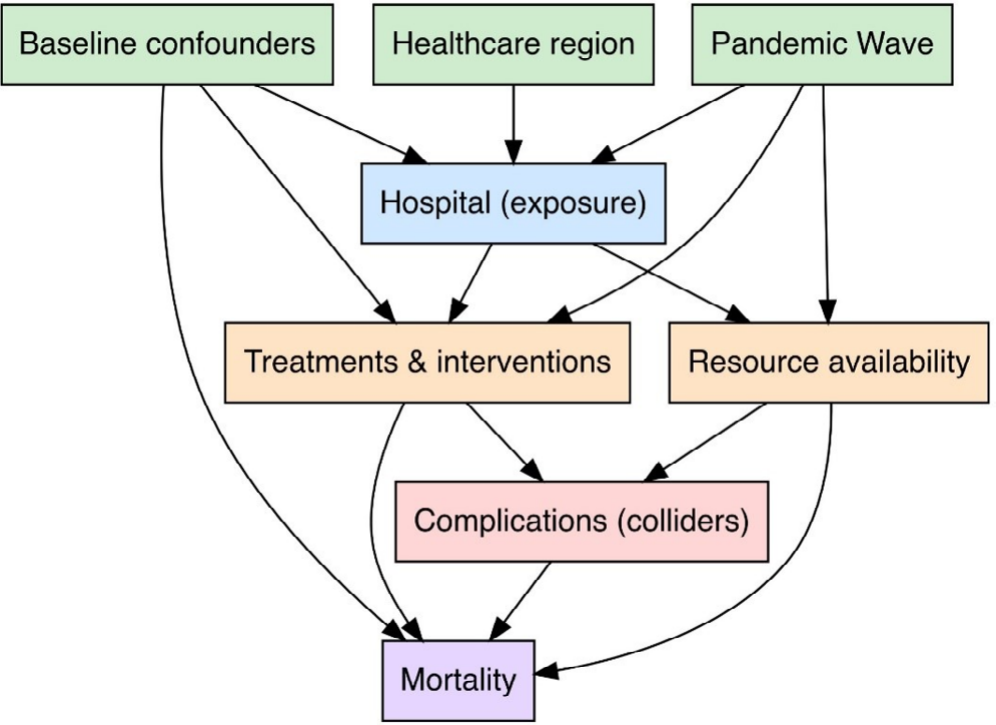
Directed acyclic graph (DAG) illustrating the assumed causal structure underlying the analysis. Hospital represents the main exposure of interest, possibly influenced by baseline confounders (comorbidity burden [CCI], sex, age, BMI, smoking status, and illness severity at admission [SAPS3]), healthcare region and the pandemic wave. Treatments, interventions and resource availability act as mediators, while complications act as colliders to mortality downstream. The DAG is schematic and intended to illustrate plausible causal structures; variables downstream of the exposure represent potential explanatory pathways and are not assumed to be measured or adjusted for within the primary analysis.

To create a conceptual causal framework to guide confounder selection for the primary analysis of exposure, hospital at ICU admission and outcome (90‐day mortality), a directed acyclic graph (DAG) was constructed (Figure 1). The DAG was constructed based on established principles of causal inference in epidemiology, including prior knowledge of relationships between baseline characteristics, care processes, and outcomes [16].

#### 2.2.8 Survival Modeling

The primary analysis used a Cox proportional hazards model with mixed effects, including a random intercept for healthcare county to account for unknown correlations within each county. To account for non‐linear time‐trends in case‐mix and clinical practice during the study period, calendar time was modeled as a continuous variable using natural cubic splines with internal knots placed on July 1, 2020, and February 16, 2021. Knot placement was decided based on local incidence peaks, corresponding to three pandemic waves. The model estimated the association between hospital of initial ICU admission (fixed effect) and 90‐day mortality, adjusting for all covariates identified within the DAG. SAPS3 was entered as a linear variable within the model. Standardized 90‐day mortality was estimated by model‐based marginal standardization (g‐computation) from fixed‐effects Cox models refitted within each imputed dataset and pooled using Rubin's rules. The resulting risks represent the predicted probability of death under hypothetical allocation of the cohort to each hospital, conditional on measured baseline covariates. Results from the survival analyses are presented as hazard ratios (95% CI) for the hospital of initial admission. For the healthcare county variable, healthcare county 2 was used as reference. For the hospital variable, Hospital B2 (the site with the lowest 90‐day mortality) was used as reference. To assess potential overfitting, events per variable (EPV) were calculated for both primary and secondary exploratory models by dividing the number of deaths at 90 days by the number of included regression coefficients.

#### 2.2.9 Missing Data

For variables included within the multivariable survival analysis, missingness was evaluated using graphical displays, correlation matrices of missing indicators, and logistic regression models to assess whether data were missing completely at random (MCAR) or at random (MAR). In cases of a substantial proportion of missing values, variables were imputed under the MAR assumption using multiple imputation by chained equations (mice package in R). For continuous variables, predictive mean matching was used. For binary variables, logistic regression was used. The imputation model included all variables in the final Cox model as predictors, together with calendar‐time splines, hospitals, and healthcare counties. Specifically, the imputation model for variable “CCI” included age, sex, smoking status, SAPS3, BMI, hospital (exposure), healthcare region, calendar‐time splines, and 90‐day mortality. Thirty imputed datasets were generated and combined using Rubin's rules. Complete case analysis is included as a sensitivity analysis. Further, a complementary sensitivity model identical to the primary analysis but excluding all transferred patients was performed.

#### 2.2.10 Secondary Analysis

In addition to our primary model estimating the association between hospital of initial ICU admission and 90‐day mortality, two secondary exploratory analyses including selected post‐exposure treatment‐related variables were conducted. Due to limited sample size at most three variables were feasible to include: inter‐hospital transfer, treatment restriction, time to intubation, and time to corticosteroid initiation. These were identified a priori and informed by descriptive differences between included hospitals. The analysis was not a formal mediation analysis and cannot distinguish causality, given both time‐dependent confounding and shared determinants of treatment timing and outcome. The purpose was to explore if inclusion of selected downstream factors attenuated the observed hospital‐level association in our primary analysis. In a separate model, treatment restrictions as a binary variable were included for explorative purposes. As the timing of treatment restrictions were lacking in our dataset, it may have occurred both upstream and downstream of our exposure of interest (hospital). Accordingly, results from the secondary analyses should only be interpreted as hypothesis‐generating. Further methodological details can be found in the Supporting Information.

## 3 Results

### 3.1 Cohort Characteristics

**FIGURE 2:**
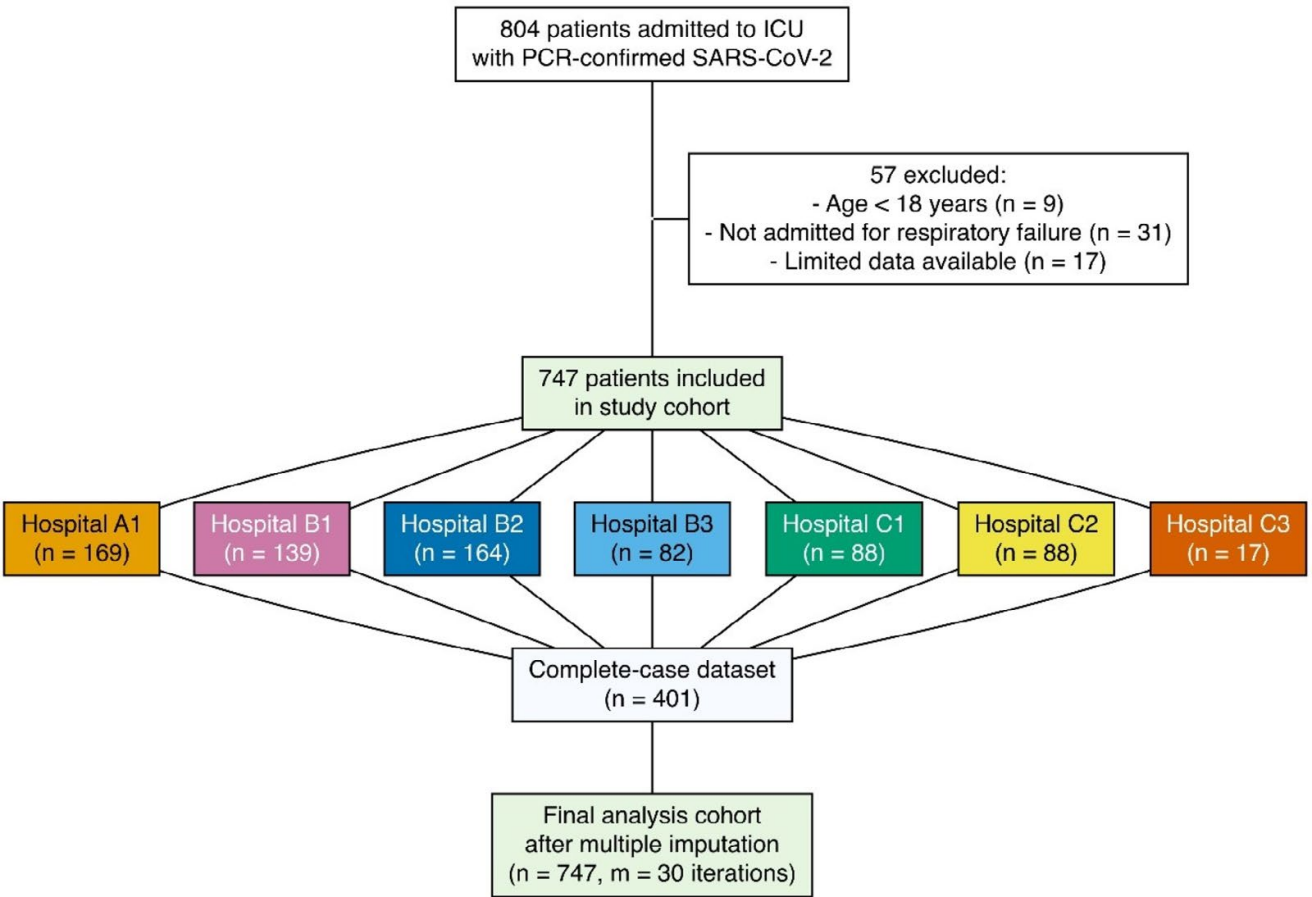
Study flowchart. All patients admitted to an ICU with PCR‐confirmed SARS‐CoV‐2 were considered for inclusion. Patients not admitted due to respiratory failure were excluded. Limited data available were due to transfers with incomplete medical journal registrations outside included healthcare counties.

**FIGURE 3:**
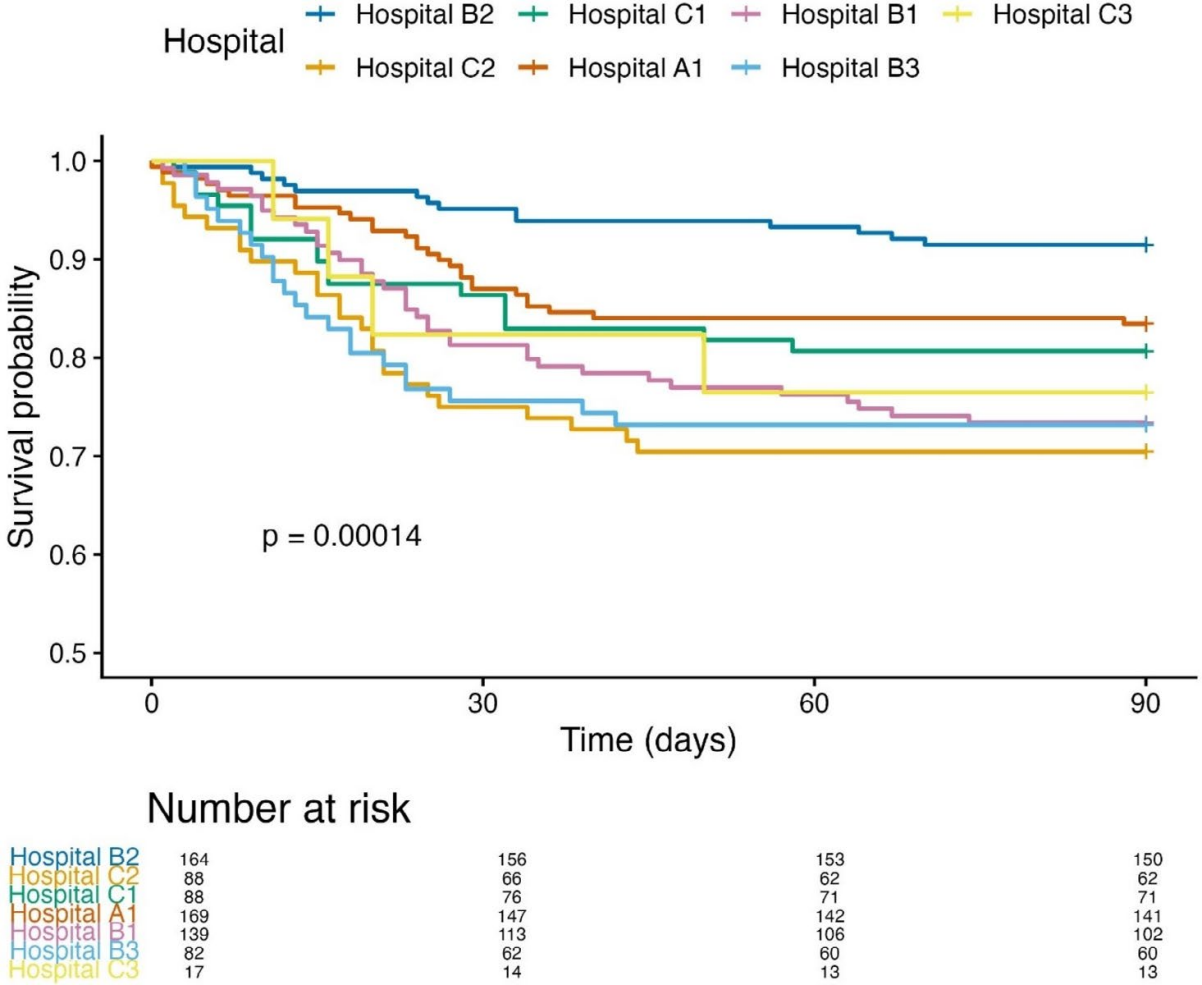
Kaplan‐Meier curve of un‐adjusted 90‐day survival of all included patients, stratified by hospital. A significant difference between the hospitals was seen with a *p*‐value of 0.00014, as tested by a log‐rank test. Y‐axis break at 0.5.

Eight hundred and four patients were identified, of which a total of 747 patients were included. A study flowchart is presented in Figure 2. Cohort characteristics and outcomes, stratified by hospital within each healthcare county, are shown in Table 1. Comparing each individual hospital, there was a difference in 90‐day mortality, with C2 having the highest 90‐day mortality (30%) and B2 the lowest (8.5%). This is also displayed in a Kaplan‐Meier curve in Figure 3. No differences regarding age, sex, and BMI were seen.

**TABLE 1:** Cohort characteristics and outcomes stratified across county and hospital.

Characteristic	*N*	Total cohort	County 1		County 2			County 3	
			Hospital A1 (*n* = 169)	Hospital B1 (*n* = 139)	Hospital B2 (*n* = 164)	Hospital C1 (*n* = 88)	Hospital C2 (*n* = 88)	Hospital B3 (*n* = 82)	Hospital C3 (*n* = 17)
90‐day mortality	747	148 (20%)	28 (17%)	37 (27%)	14 (8.5%)	17 (19%)	26 (30%)	22 (27%)	4 (24%)
Female sex	747	218 (29%)	50 (30%)	37 (27%)	53 (32%)	20 (23%)	30 (34%)	25 (30%)	3 (18%)
Current or previous smoker	546	207 (38%)	68 (40%)	69 (50%)	14 (23%)	13 (29%)	8 (24%)	28 (34%)	7 (41%)
BMI	724	30.8 (6.2)	31.0 (7.1)	30.2 (5.1)	30.4 (5.2)	30.5 (6.6)	31.6 (7.2)	31.3 (5.9)	31.8 (6.7)
Age (years)	747	62 (13)	61 (12)	64 (13)	61 (14)	62 (15)	63 (13)	60 (13)	57 (9)
Charlson comorbidity index (points)	617	1.38 (1.74)	1.13 (1.56)	0.96 (1.40)	1.60 (1.81)	1.49 (2.08)	1.86 (1.91)	1.27 (1.45)	1.00 (1.61)
SAPS3 score (points)	719	55 (10)	53 (9)	55 (11)	55 (10)	58 (11)	59 (11)	53 (8)	54 (7)
Readmitted to ICU	743	57 (7.7%)	15 (8.9%)	8 (5.9%)	21 (13%)	6 (6.8%)	0 (0%)	6 (7.4%)	1 (5.9%)
Ventilator‐free days within 90‐days	735	63 (34)	63 (33)	58 (37)	70 (26)	65 (34)	56 (39)	61 (38)	65 (37)
ARDS	525								
Mild		32 (6.1%)	3 (2.5%)	1 (1.0%)	12 (8.6%)	9 (18%)	6 (9.8%)	1 (2.4%)	0 (0%)
Moderate		223 (42%)	30 (25%)	9 (8.7%)	110 (79%)	30 (60%)	37 (61%)	6 (15%)	1 (9.1%)
Severe		270 (51%)	86 (72%)	94 (90%)	17 (12%)	11 (22%)	18 (30%)	34 (83%)	10 (91%)

*Note:* Data presented as *n* (%) and mean (±SD). Descriptive table; no hypothesis testing performed.

Several variations between included hospitals were identified. CCI ranged from 0.96, ±SD 1.4 in B1 to 1.86, ±SD 1.91 in C2. Readmissions to ICU were most common in B2 (13%) and A1 (8.9%), while no patients were readmitted at hospital C2. Further, the degree of ARDS was severe for most patients in county 1 and 3, while moderate in all hospitals within county 2.

Interventions, treatments, and complications at each hospital are presented in Table 2. Treatment restrictions were most common for patients at C2 (34%), while B2 restricted treatment the least at 11%. Tracheostomy was most frequent at B1 (81%), ranging to the lowest frequency of 27% at C3. Further, all hospitals in county 2 used less NIV before intubation. Hospital B2 and C1 used more muscle relaxants (74% and 51%, respectively). Considering treatments, a majority of patients at all hospitals were given corticosteroids; Tocilizumab and remdesivir, however, were used most in county 1, see Table 2.

**TABLE 2:** Interventions, treatments, and complications stratified across county and hospital.

Characteristic	N	Total cohort	County 1		County 2			County 3	
			Hospital A1	Hospital B1	Hospital B2	Hospital C1	Hospital C2	Hospital B3	Hospital C3
Days in hospital before ICU admission	745	3.1 (4.1)	2.9 (2.8)	3.1 (4.1)	3.5 (4.5)	3.7 (5.3)	2.5 (3.7)	2.5 (3.3)	4.4 (6.8)
Treatment restriction	747	142 (19%)	21 (12%)	37 (27%)	18 (11%)	14 (16%)	30 (34%)	19 (23%)	3 (18%)
Transferred to another ICU	747	141 (19%)	19 (11%)	16 (12%)	57 (35%)	21 (24%)	14 (16%)	7 (8.5%)	7 (41%)
Invasive mechanical ventilation	747	551 (74%)	133 (79%)	107 (77%)	142 (87%)	54 (61%)	63 (72%)	41 (50%)	11 (65%)
Days from symptom to intubation	536	11.7 (6.1)	12.2 (6.5)	12.9 (6.8)	10.7 (5.4)	10.0 (4.6)	10.6 (3.6)	12.8 (5.8)	18.3 (13.1)
Days of invasive mechanical ventilation	552	18 (18)	20 (19)	19 (15)	17 (24)	15 (10)	15 (13)	16 (15)	12 (10)
High‐flow nasal oxygen	743	601 (81%)	158 (94%)	123 (88%)	114 (70%)	57 (66%)	63 (72%)	70 (85%)	16 (94%)
Non‐invasive ventilation	741	306 (41%)	120 (72%)	88 (64%)	7 (4.8%)	20 (23%)	8 (9%)	52 (63%)	11 (65%)
Tracheostomy	551	316 (57%)	53 (40%)	87 (81%)	93 (65%)	31 (57%)	26 (41%)	23 (56%)	3 (27%)
Prone position ventilation	551	324 (59%)	36 (27%)	82 (77%)	106 (75%)	38 (70%)	30 (48%)	27 (66%)	5 (45%)
Neuromuscular block for more than 24 h	357	172 (48%)	10 (29%)	10 (25%)	104 (74%)	27 (51%)	17 (27%)	4 (18%)	0 (0%)
Renal replacement therapy	745	113 (15%)	30 (18%)	22 (16%)	25 (15%)	7 (8.0%)	16 (18%)	11 (13%)	2 (12%)
Renal replacement therapy 90 days after admission	747	7 (1%)	3 (2%)	4 (3%)	0 (0%)	0 (0%)	0 (0%)	0 (0%)	0 (0%)
Anticoagulant therapy	733	715 (98%)	164 (98%)	125 (97%)	160 (98%)	84 (97%)	84 (97%)	81 (99%)	17 (100%)
Remdesivir	742	110 (15%)	47 (28%)	26 (19%)	14 (8.5%)	6 (6.9%)	7 (8.0%)	9 (11%)	1 (5.9%)
Hydroxy‐chloroquine	743	13 (2%)	8 (5%)	0 (0%)	0 (0%)	0 (0%)	1 (1%)	4 (5%)	0 (0%)
Chloroquine	743	18 (2%)	0 (0%)	10 (7%)	5 (3%)	1 (1%)	1 (1%)	1 (1%)	0 (0%)
Tocilizumab	743	79 (11%)	23 (14%)	32 (23%)	1 (0.6%)	2 (2%)	7 (8%)	14 (17%)	0 (0%)
Anakinra	743	1 (0.1%)	1 (0.6%)	0 (0%)	0 (0%)	0 (0%)	0 (0%)	0 (0%)	0 (0%)
Corticosteroid treatment	742	624 (84%)	128 (77%)	109 (80%)	146 (89%)	75 (86%)	76 (86%)	75 (91%)	15 (88%)

*Note:* Data presented as *n* (%) and mean (±SD). Descriptive table; no hypothesis testing performed.

### 3.2 Primary Analysis

In the mixed‐effects Cox model adjusting for baseline characteristics, disease severity, and calendar time, the initial hospital of admission was significantly associated with 90‐day mortality. Hazard ratios ranged from 2.38 to 5.06 (Table 3). The corresponding standardized 90‐day mortality ranged from 8.3% to 31.8% (Table 4). For the primary model, the EPV was 9.9, indicating acceptable model stability. Coefficients for all adjustment factors included in the model can be found in the supplement quarto file “Survival analysis”.

**TABLE 3:** Primary analysis of inter‐hospital differences in 90‐day mortality.

Variable	Hazard ratio (95% CI)	*p*	*E*
Hospital A1	2.52 (1.31, 4.83)	**0.005**	3.18
Hospital B1	3.62 (1.92, 6.82)	**< 0.001**	4.23
Hospital B2	1	—	—
Hospital B3	5.06 (2.56, 10.02)	**0.007**	5.40
Hospital C1	2.38 (1.16, 4.89)	**0.018**	3.03
Hospital C2	3.85 (1.98, 7.48)	**< 0.001**	4.43
Hospital C3	4.75 (1.53, 14.74)	**0.007**	5.17

*Note:* Hazard ratios (95% CI) derived from random‐effects Cox regression with healthcare county as random intercept and initial hospital of ICU admission, pandemic wave, and baseline covariates as fixed effects. Analyses performed on multiply imputed data (*m* = 30) and pooled using Rubin's rules. Hazard ratio > 1 indicates higher mortality relative to reference Hospital B2. *N* = 747. Baseline covariates were CCI, SAPS3 upon ICU admission, age, sex, smoking status, and BMI. For pandemic wave, three splines with two internal knots at July 1, 2020 and February 16, 2021 was used. Bold *p*‐values highlights significance (*p* < 0.05).

**TABLE 4:** Adjusted 90‐day mortality estimated by marginal standardization of model‐based predictions.

Variable	Standardized 90‐day mortality (%)
Hospital A1	18.6
Hospital B1	24.9
Hospital B2	8.3
Hospital B3	31.8
Hospital C1	17.7
Hospital C2	25.8
Hospital C3	30.4

*Note:* Models were adjusted for baseline covariates and pandemic wave. Estimates were combined across imputations (*m* = 30) using Rubin's rules. The mortality represents the predicted risk if all patients in the cohort had been treated at that hospital. Baseline covariates were CCI, SAPS3 upon ICU admission, age, sex, smoking status, and BMI. For pandemic wave, three splines with two internal knots at July 1, 2020 and February 16, 2021 was used.

### 3.3 Sensitivity Analysis and Robustness

Complete‐case analysis (*n* = 401) yielded effect estimates that were consistent with the imputed main model, but with wider confidence intervals and two hospital effects lost nominal statistical significance (hospitals A1 and C2). Results from the complete case analysis can be found in supplementary Table S1. Between‐county heterogeneity was small (random intercept SD 0.04), indicating minimal between‐county variability. E‐value analysis indicated that an unmeasured confounder would need an association to both hospital and mortality with a risk ratio of 3–5 to fully explain the observed differences (Table 3). No multi‐collinearity was identified, and the assumption of proportional hazards was met (see supplementary quarto file “Survival analysis”).

A total of 141 (19%) of the cohort was transferred to another ICU. Hospital C3 and B2 transferred most patients (41% and 35%, respectively), while A1 and B1 transferred patients to a lesser extent (11% and 12% transfers, respectively). Comparing transferred to non‐transferred patients, no significant difference in 90‐day mortality was seen (17% and 20%, respectively). Further, no difference was seen in sex, age, or SAPS3 at admission (data not shown). Transferred patients had a lower CCI (1.27 [1.51]) compared to non‐transferred (1.41 [1.80]), and treatment restrictions were less common (11% vs. 21%). Further, a majority of transferred patients were in IMV (91%) compared to non‐transfers (70%). Running a complementary sensitivity model excluding transferred patients reduced the sample size and number of events (EPV ≈8), and resulted in wider confidence intervals. Directionally similar results were seen, but hospital C1 and C3 lost nominal significance. Results from this analysis can be found in Table S2.

### 3.4 Missing Data and Imputation

Missingness was modest for most covariates (below 4%), except for smoking status and CCI (27% and 17%, respectively). Logistic regressions displayed that missingness was associated with sex and hospital for smoking status, and with hospital and SAPS3 for CCI, supporting that data was missing at random (MAR). Please see Missing analysis in Supporting Information for further details. Due to this, missing values were imputed using chained equations (creating 30 datasets) with predictive mean matching for continuous variables and logistic regression for binary variables. Multiple imputation yielded stable convergence (see trace plots, Survival analysis in Supporting information), and the mean fraction of missing information was below 9% for all variables.

### 3.5 Secondary Analysis

The exploratory secondary model included the same baseline covariates as the primary analysis, with the addition of three treatment‐related variables downstream to the exposure hospital (transfer, time to intubation, and time to corticosteroid initiation). Data were imputed (creating 30 datasets) and analyzed as time‐dependent Cox models with pooling by Rubin's rules (Table S3). EPV in the exploratory model was 8.2, indicating a risk of overfitting, with wide confidence intervals for several hospitals. The hospital variable block remained highly significant in the pooled Wald test (*χ* ^2^ = 35.98 df = 6, *p* < 0.001), indicating persistent contextual mortality differences between hospitals after adjustment for the included treatment‐related factors. An additional exploratory model including all variables within our primary analysis with the addition of treatment restrictions was conducted; results were slightly attenuated but directionally similar (Table S4). All R code for the secondary analysis is available in supplementary quarto file named “secondary analysis” and “treatment restriction”.

## 4 Discussion

In this multicenter cohort study of critically ill COVID‐19 patients, we present significant differences in 90‐day mortality between the hospitals at which patients were initially admitted, irrespective of subsequent inter‐hospital transfers. One hospital presented with significantly lower 90‐day mortality than all other included units. The increased hazard persisted after adjustment for baseline confounders, calendar time, and healthcare county. The results were robust across sensitivity analyses and indicate a contextual association related to hospital of initial ICU admission, possibly reflecting a combination of organizational characteristics, clinical routines, ICU strain, and admission thresholds that are not captured in the current dataset.

The observed inter‐hospital differences should not be interpreted as a direct reflection of quality of care, competence, or the effectiveness of specific interventions at individual units. Nor do the results imply that variations in treatment strategies—such as respiratory care or pharmacological timing—can be causally linked to the observed mortality variation. Given the contextual nature of our defined exposure and absence of detailed organizational and ICU selection related data, attributing the observed association to specific care processes or clinical decisions risks over‐interpretation.

Substantial inter‐hospital variations in mortality were nevertheless found and persisted through adjustment and sensitivity analyses, raising the question of what underlies these differences. In the present study, the exposure “hospital of initial ICU admission” represents a contextual construct and likely captures unmeasured mediators not available within the dataset. Plausible explanations may be defined as (1) treatment‐related at a patient‐level, such as timing of certain interventions, treatments used and care routines—and (2) organizational factors (strain, staffing levels, patient admission surges). Differences in care patterns were seen, as Hospital B2 used less NIV, intubated early, used more muscle relaxants, had longer durations of IMV, transferred more patients, readmitted more often, and had the lowest degree of treatment restrictions. These factors suggest a difference in respiratory management strategy and possibly a higher threshold for restricting intensive care. However, current evidence does not clearly support that such differences in respiratory management translate into mortality differences [17, 18, 19]. NIV may be appropriate for a subset of patients with mild ARDS [17], but its use must be balanced against the risk of patient self‐inflicted lung injury [18], and no clear consensus exists regarding timing of intubation [19]. Additionally, transfers between hospitals often necessitated intubation—influencing both who was transferred and timing of intubation. Further, including a few key (transfers, time to intubation, and time to corticosteroid treatment) variables within our secondary exploratory analysis did not materially change outcomes.

The decision to restrict treatments will, by definition, impact mortality. During the pandemic, treatment restrictions within ICUs were widely debated in the scientific community [20, 21]. As COVID‐19 was a novel disease, the prognostication in critically ill patients were particularly challenging during the study period. In the current study, treatment restriction was registered as a dichotomous variable which restricted more detailed analyses of its timing, scope, and clinical context. The hospital “culture” regarding treatment limitations likely acts as an unmeasured confounder in our dataset, as restricting care may have influenced our cohort composition through ICU admission thresholds. ICUs that admit patients with more advanced comorbidity or frailty might have higher mortality due to case‐mix differences not fully captured in adjusted analyses. Particularly during periods of high ICU strain admission practices might change [9], and as the current study lacks data on non‐ICU COVID patients, such cohort differences remain unaccounted for. Supporting this, a US study of patients with pneumonia found that, if not accounted for, mortality may be over‐estimated in hospitals with higher rates of treatment restrictions [22]. The underlying reasons behind decisions to limit treatment are complex and difficult to fully capture. Factors such as staffing levels, clinician's experience and continuity, patient inflow and ICU capacity may have influence, even though they ideally should not. Within our exploratory model including treatment restrictions, results were attenuated but still statistically significant.

Suggesting that treatment restrictions may contribute to, but are unlikely to fully account for, the observed mortality differences. As treatment restrictions may reflect both pre‐ICU selection and decisions made during the ICU stay, even the adjusted analysis cannot determine whether treatment restrictions acted as a selection mechanism, mediator, marker of prognosis, or downstream treatment decision.

Comparisons between ICUs may also be influenced by availability of advanced respiratory support outside of the ICU. Certain hospitals had greater access to non‐invasive respiratory support at non‐ICU levels, which might explain why so few ICU patients in B3 were intubated as compared with the other hospitals. This is a common challenge when comparing different ICU cohorts, particularly across different healthcare systems [23], as organizational structures and admission practices vary.

Taken together, a definitive explanation for the observed differences cannot be established within the current study. Likely, hospital of initial ICU admission summarizes the effect of differences at an organizational level. Inter‐hospital variation in ICU strain, capacity, patient‐to‐nurse ratios, admission surges, and admission thresholds are plausible contributors and are well‐established determinants of ICU outcomes [5, 6, 7, 24]. Larger datasets incorporating detailed information on organizational level factors such as staffing, resource availability, ICU strain, admission surges, and admission thresholds are needed to elucidate the mechanisms driving these mortality variations, and to identify care components amenable to improvement.

The Public Health Agency of Sweden (PHAS) has issued eight national public health objectives, where healthcare equity is one [25]. In addition, Sweden's healthcare counties have collaboratively established 26 National Program Areas, with the overarching objective of promoting equitable and knowledge‐based healthcare delivery [26]. Apart from contextual differences, the observed inter‐hospital mortality differences in our material could suggest that the care provided to COVID‐19 patients admitted to an ICU may not have been consistent across hospitals—raising concerns about equality in care delivery during the pandemic. These findings underscore the importance of exploring hospital‐level variations in care related to organizational or treatment factors. Systematically sharing practices between units, particularly when facing new diseases where management pathways may diverge, is essential.

## 5 Limitations

There are several limitations to this study. Firstly, as treatment restriction before ICU admission/admission thresholds, at least one baseline confounder was not included in the Cox model. However, calculated E‐values suggest that an unmeasured confounder would need to have a strong association with both the exposure and the outcome to dismiss the observed associations. But, *E*‐values are simply calculated from the hazard ratio and its confidence interval, and they should be interpreted in relation to all other results presented.

Secondly, differences in documentation routines may lead to misclassification or systematic errors. As retrospective studies rely on previously recorded data, missingness is unavoidable. The observed differences between the multiple imputed and complete‐case analyses likely reflect loss of statistical power, as the complete case model excluded roughly 45% of the cohort. However, the directionally consistent estimates across imputed and complete‐case analyses support the stability and robustness of our imputation approach.

Thirdly, one could argue whether patients transferred between hospitals should have been excluded. Excluding them would have resulted in loss of patients from the analysis, thereby reducing statistical power. This was seen within our sensitivity model excluding transferred patients, with EPV of 8.2 and wider confidence intervals. Including transfers enables evaluation of the full course of ICU patient management. This includes the effect of transfers, but diminishes the impact of the ICU care, especially when the patients were transferred early in their ICU course and received most of their care at another hospital. Therefore, the available data do not always reflect where these patients were primarily treated if they underwent one or more transfers. But our sensitivity model excluding all transferred patients did not indicate major differences regarding the direction of the primary model results.

Fourthly, data on staffing levels and ICU strain were unavailable. While these factors are unlikely to represent baseline patient‐level confounders in our primary analysis, their absence limits our ability to explore potential mechanisms underlying observed hospital‐level differences.

Fifth, our analyses specifically focus on the pre‐Omicron phase of the pandemic. Although vaccination programs were gradually implemented and various SARS‐CoV‐2 variants circulated during the study period, we lacked sufficiently detailed or complete data on individual vaccination status and viral variant to explicitly account for these factors.

Lastly, since differences in referral patterns, ICU organization, case‐mix, pandemic burden, and resource availability may influence both mortality and inter‐hospital variation, our results may not be directly generalizable to other Swedish or Scandinavian ICUs.

## 6 Conclusion

Among patients admitted to ICU due to COVID‐19, we observed a difference in mortality related to the hospital of first‐ICU admission. This difference persisted after adjustment for calendar time, baseline confounders, and healthcare county. Potential explanations are lacking within the current study. Future studies should focus on a comprehensive evaluation of both organizational and contextual determinants of mortality.

## Author Contributions

All authors of this manuscript follow the ICMJE (international committee of medical journal editors) definition of authors. Study design was made primarily by J.I., H.J., and G.F. with contribution from all authors. G.F. wrote the initial manuscript draft, and subsequent versions were written with contribution from all authors. G.F. conducted all descriptive statistics and the uni‐and multivariable Cox analysis. Ethical approval was obtained by L.E. and J.I. Funding was secured by G.F., L.E., and J.I. Final version was read and approved by all authors.

## Funding

The study was funded by Region Östergötland (ref 978201, 1005642) and the Medical Research Council of Southeast Sweden (ref FORSS‐941384, ‐964312, ‐968674, ‐1013279). Clinical Studies Sweden/Swedish Research Council 2022.

## Conflicts of Interest

The authors declare no conflicts of interest.

## Supporting information


**Data S1:** Missing analysis.

**Data S2:** Direct effect analysis.

**Data S3:** Survival analysis and sensitivity testing.

**Data S4:** Treatment restrictions.

**Data S5:** STROBE statement—checklist of items that should be included in reports of observational studies.

**Data S6:** Supplementary methods.

**Table S1:** Complete case random‐effects Cox regression model (*n* = 401) analyzing inter‐hospital differences in 90‐day mortality. Random intercept for healthcare county. Initial hospital of ICU admission, pandemic wave and baseline covariates as fixed effects.

**Table S2:** Complementary random‐effects cox regression sensitivity model excluding all transferred patient (*n* = 606). Random intercept for healthcare county. Initial hospital of ICU admission, pandemic wave and baseline covariates as fixed effects. Analyses performed on multiply imputed data (*m* = 30) and pooled using Rubin's rules.

**Table S3:** Exploratory time‐dependent Cox regression including treatment‐related variables, baseline covariates, initial hospital of ICU admission and pandemic wave. Analyses performed on multiply imputed data (*m* = 30) and pooled using Rubin's rules.

**Table S4:** Exploratory time‐dependent Cox regression including treatment restriction in addition to baseline covariates, initial hospital of ICU admission and pandemic wave. Analyses performed on multiply imputed data (*m* = 30) and pooled using Rubin's rules.

## Data Availability

The data that support the findings of this study are available from the corresponding author upon reasonable request.
