# Supplementary material for: COVID‐19 Mortality in Swedish Intensive Care Units: A Multicenter Survival Analysis
Source: Acta Anaesthesiol Scand. 2026 Jun 14;70(6):e70279. doi: 10.1111/aas.70279 (PMC13265249; doi:10.1111/aas.70279)
Supplement: Supplementary file 1 — Data S1: Missing analysis. [file AAS-70-0-s006.pdf]

missing analysis

Gustaf Forsberg

2026-02-12

Below, relevant packages are loaded, data is loaded and prepared for analysis

```
library(pacman)
p_load(readxl, MissMech, naniar, VIM, mice, dplyr, tidyr, ggplot2, broom)
my_data <- read_excel("descriptive_mort_v3.xlsx", sheet = "Blad3")
my_data <- my_data %>%
  mutate(
    Sjukhus = factor(Sjukhus,
                     levels = 1:7,
                     labels = c("Hospital B2", "Hospital C2", "Hospital C1", "Hospital
                      ↪  A1", "Hospital B1", "Hospital B3", "Hospital C3")),
    Sjukvardsregion = factor(Sjukvardsregion,
                             levels = 1:3,
                             labels = c("Region 1", "Region 2", "Region 3")),
    BMI = as.numeric(BMI)
  )

df <- my_data

char_cols <- names(df)[vapply(df, is.character, TRUE)]
if (length(char_cols)) df[char_cols] <- lapply(df[char_cols], factor)
```

Below, missing data is visualized

```
p_load(stringr)

vars_plot <- c(
  "Ninety_day_mortality","Tid_censur_event",
  "Age","Woman","BMI","CCI","SAPS3",
  "Current_or_x_smoker",
  "Sjukhus","Sjukvardsregion",
  "Transfer_within_hospital_region"
  # lägg till fler om du vill, men håll det rimligt
)

df_miss <- df %>%
  select(any_of(vars_plot)) %>%
  rename(
    `90d mortality` = Ninety_day_mortality,
    `Time to event` = Tid_censur_event,
    `Female`        = Woman,
```

```
    `Smoking`        = Current_or_x_smoker,
    `Hospital`       = Sjukhus,
    `Region`         = Sjukvardsregion,
    `Transfer`       = Transfer_within_hospital_region
  )

library(naniar)
library(ggplot2)

#| fig-width: 10
#| fig-height: 5
#| fig-cap: "Missingness heatmap (selected variables)"

p1 <- vis_miss(df_miss, sort_miss = TRUE, cluster = TRUE) +
  theme_minimal(base_size = 12) +
  theme(
    axis.text.x = element_text(angle = 45, hjust = 1),
    panel.grid = element_blank(),
    legend.position = "bottom"
  )

p1
```

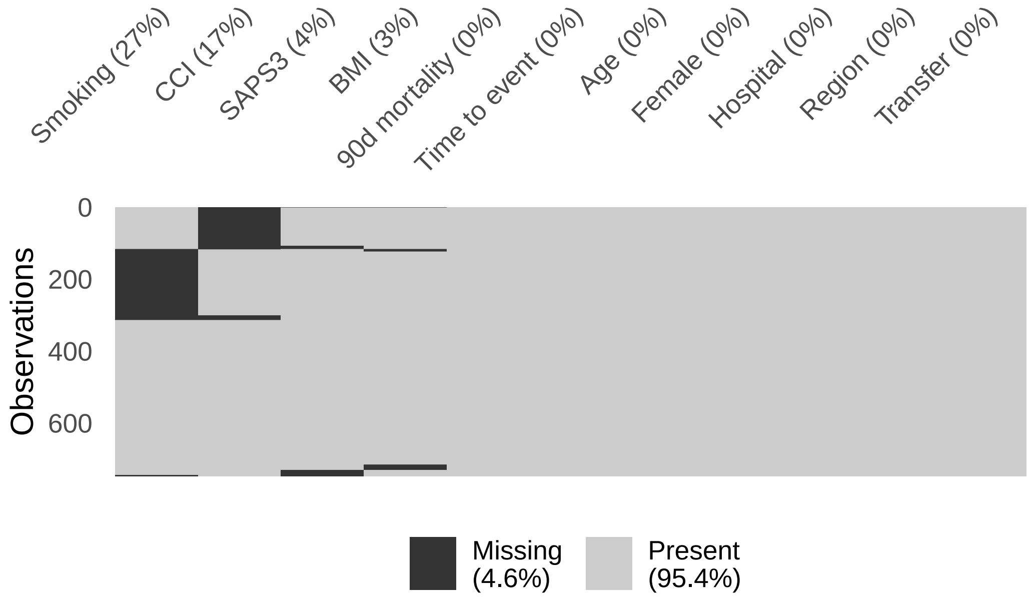


```
library(naniar)
library(ggplot2)

#| fig-width: 9
#| fig-height: 4
gg_miss_var(df_miss, show_pct = TRUE) +
  theme_minimal(base_size = 12) +
  theme(axis.text.x = element_text(angle = 45, hjust = 1))
```

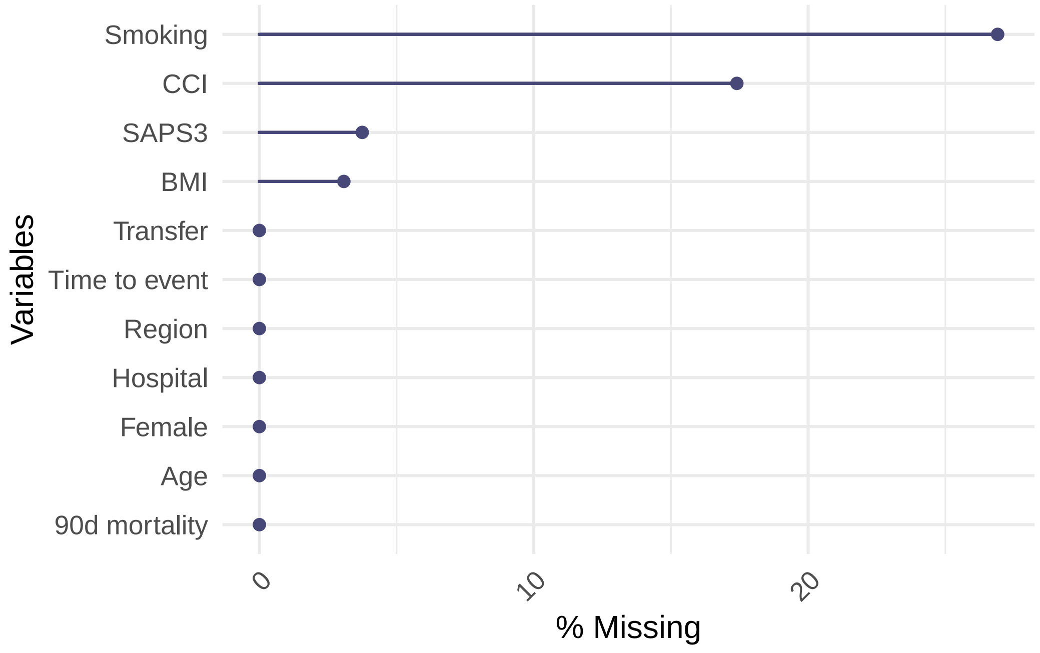

Below, logistic regression models for the two variables with highest degree of missing (CCI and smoking status) are built to determine correlation with other variables (MAR and not MCAR)

```r
check_mar_robust <- function(var) {
  d <- my_data %>%
    mutate(
      miss = as.integer(is.na(.data[[var]])),
      Age_imp   = ifelse(is.na(Age), median(Age, na.rm=TRUE), Age),
      SAPS3_imp = ifelse(is.na(SAPS3), median(SAPS3, na.rm=TRUE), SAPS3),
      Woman_fac = factor(Woman),
      Sjukhus_fac = factor(Sjukhus),
      Region_fac  = factor(Sjukvardsregion)
    )
  f <- miss ~ Age_imp + SAPS3_imp + Woman_fac + Sjukhus_fac + Region_fac
  d <- d[!is.na(d$miss), ]
  if (nrow(d) > 50 && length(unique(d$miss)) == 2) {
    broom::tidy(glm(f, data=d, family=binomial()))
  } else NULL
}

mar_smoker <- check_mar_robust("Current_or_x_smoker")
mar_cci    <- check_mar_robust("CCI")
mar_smoker; mar_cci
```

```
# A tibble: 12 x 5
   term                    estimate   std.error   statistic       p.value
   <chr>                      <dbl>       <dbl>       <dbl>         <dbl>
 1 (Intercept)             -0.111       0.634   -0.176       0.861
 2 Age_imp                  0.00972     0.00960  1.01        0.311
 3 SAPS3_imp                0.00417     0.0127   0.328       0.743
 4 Woman_fac1              -0.562       0.243   -2.31        0.0206
 5 Sjukhus_facHospital C2  -0.0832      0.278   -0.299       0.765
```

```
 6 Sjukhus_facHospital C1  -0.650       0.275   -2.37        0.0180
 7 Sjukhus_facHospital A1  -5.70        1.02    -5.60        0.0000000210
 8 Sjukhus_facHospital B1 -24.5      7765.      -0.00315     0.997
 9 Sjukhus_facHospital B3 -22.1     30322.      -0.000728    0.999
10 Sjukhus_facHospital C3 -22.1     29259.      -0.000756    0.999
11 Region_facRegion 2      -0.0448  30151.      -0.00000149  1.00
12 Region_facRegion 3      NA          NA       NA          NA

# A tibble: 12 x 5
   term                   estimate std.error statistic     p.value
   <chr>                     <dbl>     <dbl>     <dbl>       <dbl>
 1 (Intercept)            -1.34      0.873     -1.54    0.124
 2 Age_imp                -0.00691   0.00976   -0.708   0.479
 3 SAPS3_imp              -0.0406    0.0144    -2.81    0.00496
 4 Woman_fac1             -0.250     0.245     -1.02    0.306
 5 Sjukhus_facHospital C2  0.373     0.926      0.403   0.687
 6 Sjukhus_facHospital C1  2.03      0.677      3.00    0.00270
 7 Sjukhus_facHospital A1  2.63      0.615      4.28    0.0000187
 8 Sjukhus_facHospital B1  2.82      0.621      4.54    0.00000574
 9 Sjukhus_facHospital B3 -9.12    535.        -0.0170  0.986
10 Sjukhus_facHospital C3 -9.69    535.        -0.0181  0.986
11 Region_facRegion 2     13.1     535.         0.0245  0.980
12 Region_facRegion 3     NA        NA         NA      NA
```
