# Supplementary material for: COVID‐19 Mortality in Swedish Intensive Care Units: A Multicenter Survival Analysis
Source: Acta Anaesthesiol Scand. 2026 Jun 14;70(6):e70279. doi: 10.1111/aas.70279 (PMC13265249; doi:10.1111/aas.70279)
Supplement: Supplementary file 2 — Data S2: Direct effect analysis. [file AAS-70-0-s007.pdf]

Direct effect analysis

Gustaf Forsberg

2026-02-12

In this document, the direct effect of hospital at admission is explored. This is an exploratory analysis, with no ambition to explain causality but simply explore the established association between initial hospital of ICU admission and mortality, given adjustment for a few mediators of the hospital variable.

```
library(pacman)
p_load(readxl, MissMech, naniar, VIM, mice, dplyr, tidyr, ggplot2, broom, survival,
 ↪  lubridate, miceadds, splines, tidyr, coxme, car, stdReg)

my_data <- read_excel("descriptive_mort_v3.xlsx", sheet = "Blad4")
my_data <- my_data %>%
  mutate(
    Sjukhus = factor(Sjukhus,
                     levels = 1:7,
                     labels = c("Hospital B2", "Hospital C2", "Hospital C1", "Hospital
                      ↪  A1", "Hospital B1", "Hospital B3", "Hospital C3")),
    BMI = as.numeric(BMI)
  )


my_data$Woman <- factor(my_data$Woman, levels = c("0", "1"))
my_data$Woman <- relevel(my_data$Woman, ref = "1")
my_data$Current_or_x_smoker <- factor(my_data$Current_or_x_smoker, levels = c("0", "1"))
my_data$Current_or_x_smoker <- relevel(my_data$Current_or_x_smoker, ref = "0")
my_data$Transfer_within_hospital_region <-
 ↪  factor(my_data$Transfer_within_hospital_region, levels = c("0", "1"))
my_data$Transfer_within_hospital_region <-
 ↪  relevel(my_data$Transfer_within_hospital_region, ref = "0")


my_data$cs_treat_start[is.na(my_data$cs_treat_start)] <-
 ↪  my_data$Tid_censur_event[is.na(my_data$cs_treat_start)] + 1
my_data$days_intub_start[is.na(my_data$days_intub_start)] <-
 ↪  my_data$Tid_censur_event[is.na(my_data$days_intub_start)] + 1

my_data <- my_data %>%
  mutate(Tid_censur_event = ifelse(Tid_censur_event == 0, 0.5, Tid_censur_event))

df <- my_data

char_cols <- names(df)[vapply(df, is.character, TRUE)]
if (length(char_cols)) df[char_cols] <- lapply(df[char_cols], factor)
```

Below, splines with internal knots resembling pandemic wave 1, 2 and 3 are created

```r
df <- my_data %>%
  mutate(
    admission_date = as.Date(admission_date),
    date_num = as.numeric(admission_date),
    Ninety_day_mortality = as.integer(Ninety_day_mortality),
  )

k1 <- as.numeric(as.Date("2020-07-01"))
k2 <- as.numeric(as.Date("2021-02-16"))

NS <- ns(df$date_num, knots = c(k1, k2))
colnames(NS) <- paste0("cs_date_", seq_len(ncol(NS)))
df <- bind_cols(df, as.data.frame(NS))
```

Now, an identical multiple imputation is made for the exploratory model

```r
vars_keep <- c(
  "Tid_censur_event","Ninety_day_mortality",
  "Current_or_x_smoker","CCI","SAPS3","BMI",
  "Age","Woman","Sjukhus","Transfer_within_hospital_region",
  "cs_treat_start","days_intub_start",
  "admission_date","date_num", colnames(NS)
)

dat <- dplyr::select(df, dplyr::any_of(vars_keep))

dat$Current_or_x_smoker <- factor(dat$Current_or_x_smoker, levels = c(0,1), labels =
↪  c("No","Yes"))

meth <- make.method(dat); meth[] <- ""
meth["Current_or_x_smoker"] <- "logreg"
meth["CCI"]  <- "pmm"
meth["SAPS3"]<- "pmm"
meth["BMI"]  <- "pmm"

pred <- make.predictorMatrix(dat); pred[,] <- 0
base_preds <- setdiff(vars_keep, c("Current_or_x_smoker","CCI","SAPS3","BMI"))

pred["Current_or_x_smoker", c(base_preds,"CCI","SAPS3","BMI")] <- 1
pred["CCI",                 c(base_preds,"Current_or_x_smoker","SAPS3","BMI")] <- 1
pred["SAPS3",               c(base_preds,"Current_or_x_smoker","CCI","BMI")] <- 1
pred["BMI",                 c(base_preds,"Current_or_x_smoker","CCI","SAPS3")] <- 1

meth[c("Tid_censur_event","Ninety_day_mortality","Age","Woman","Sjukhus",
       "Transfer_within_hospital_region","cs_treat_start","days_intub_start",
       "admission_date","date_num", colnames(NS))] <- ""

m <- 30
set.seed(2025)
imp <- mice(dat, m = m, maxit = 20, method = meth, predictorMatrix = pred, printFlag =
↪  FALSE)
```

```
Warning: Number of logged events: 2401
```

Now, a time-dependent dataset is created and a coxph analysis is made on each imputed dataset and pooled

```
pool_est <- list()
pool_vcv <- list()

for (k in 1:m) {
  d_k <- complete(imp, k)


  d_k <- d_k |>
    mutate(
      id = dplyr::row_number(),
      cs_day   = ifelse(is.na(cs_treat_start),    Inf, pmax(0, cs_treat_start)),
      intu_day = ifelse(is.na(days_intub_start),  Inf, pmax(0, days_intub_start))
    )

  # Baseline tmerge: start=0, stop=Tid_censur_event, event = Ninety_day_mortality
  long_k <- tmerge(
    data1 = d_k,
    data2 = d_k,
    id    = id,
    tstart = 0,
    tstop  = Tid_censur_event
  )
  long_k <- tmerge(
    data1 = long_k,
    data2 = d_k,
    id    = id,
    death = event(Tid_censur_event, Ninety_day_mortality),
    cs_td = tdc(cs_day),
    intu_td = tdc(intu_day)
  )

  # Cox time dependent
  fit_k <- coxph(
    Surv(tstart, tstop, death) ~
      Sjukhus +
      Current_or_x_smoker + CCI + SAPS3 + BMI + Age + Woman +
      Transfer_within_hospital_region +
      cs_td + intu_td +
      cs_date_1 + cs_date_2 + cs_date_3,
    data = long_k,
    ties = "breslow",
    cluster = id
  )

  pool_est[[k]] <- coef(fit_k)
  pool_vcv[[k]] <- vcov(fit_k)
}

# Rubin-pooling
pool_rubin <- function(estimates, variances){
  m <- length(estimates)
  qbar <- Reduce("+", estimates)/m
  ubar <- Reduce("+", variances)/m
```

```r
  b <- Reduce("+", lapply(estimates, function(q) (q - qbar) %*% t(q - qbar))) / (m - 1)
  tvar <- ubar + (1 + 1/m)*b
  list(estimates = qbar, variances = tvar)
}

comb <- pool_rubin(pool_est, pool_vcv)
res  <- data.frame(
  term = names(comb$estimates),
  estimate = comb$estimates,
  se = sqrt(diag(comb$variances))
)
res$HR  <- exp(res$estimate)
res$LCL <- exp(res$estimate - 1.96*res$se)
res$UCL <- exp(res$estimate + 1.96*res$se)
res$p   <- 2*pnorm(-abs(res$estimate/res$se))
res
```

```
                                                                term    estimate
SjukhusHospital C2                                SjukhusHospital C2  1.46971782
SjukhusHospital C1                                SjukhusHospital C1  1.00368804
SjukhusHospital A1                                SjukhusHospital A1  1.00562596
SjukhusHospital B1                                SjukhusHospital B1  1.39742266
SjukhusHospital B3                                SjukhusHospital B3  1.85549975
SjukhusHospital C3                                SjukhusHospital C3  1.88643666
Current_or_x_smokerYes                        Current_or_x_smokerYes -0.06518243
CCI                                                              CCI  0.01796609
SAPS3                                                          SAPS3  0.03160129
BMI                                                              BMI -0.01246336
Age                                                              Age  0.05453061
Woman0                                                        Woman0  0.13764798
Transfer_within_hospital_region1 Transfer_within_hospital_region1 -0.03277873
cs_td                                                          cs_td  0.55594310
intu_td                                                      intu_td  0.92645130
cs_date_1                                                  cs_date_1 -0.32913860
cs_date_2                                                  cs_date_2 -2.08136575
cs_date_3                                                  cs_date_3 -0.49345630
                                         se        HR         LCL       UCL
SjukhusHospital C2               0.34739534 4.3480081 2.200804442  8.590120
SjukhusHospital C1               0.37474364 2.7283255 1.308904476  5.687015
SjukhusHospital A1               0.32985584 2.7336179 1.432052185  5.218152
SjukhusHospital B1               0.31276540 4.0447618 2.191097311  7.466623
SjukhusHospital B3               0.34007696 6.3948933 3.283628000 12.454109
SjukhusHospital C3               0.58820030 6.5958236 2.082489536 20.890808
Current_or_x_smokerYes           0.21421168 0.9368965 0.615674210  1.425714
CCI                              0.05560483 1.0181285 0.913000018  1.135362
SAPS3                            0.01162655 1.0321059 1.008852208  1.055896
BMI                              0.02092315 0.9876140 0.947931784  1.028957
Age                              0.01100173 1.0560448 1.033516652  1.079064
Woman0                           0.20553198 1.1475715 0.767056589  1.716849
Transfer_within_hospital_region1 0.24796972 0.9677527 0.595234773  1.573405
cs_td                            0.23782473 1.7435846 1.093962721  2.778968
intu_td                          0.22480884 2.5255309 1.625517082  3.923863
cs_date_1                        0.46504921 0.7195433 0.289200501  1.790255
```

```
cs_date_2                          1.54475869 0.1247597 0.006041561  2.576318
cs_date_3                          0.63438874 0.6105126 0.176072710  2.116885
                                              p
SjukhusHospital C2                 2.329880e-05
SjukhusHospital C1                 7.398974e-03
SjukhusHospital A1                 2.298467e-03
SjukhusHospital B1                 7.896983e-06
SjukhusHospital B3                 4.866587e-08
SjukhusHospital C3                 1.340649e-03
Current_or_x_smokerYes             7.609071e-01
CCI                                7.466172e-01
SAPS3                              6.567211e-03
BMI                                5.513935e-01
Age                                7.175707e-07
Woman0                             5.030391e-01
Transfer_within_hospital_region1 8.948352e-01
cs_td                              1.940713e-02
intu_td                            3.771290e-05
cs_date_1                          4.791005e-01
cs_date_2                          1.778602e-01
cs_date_3                          4.366602e-01
```

```r
## --- Model complexity: events-per-variable (EPV) and hospital block test ---

b_all <- as.numeric(comb$estimates)
V_all <- as.matrix(comb$variances)


p_df_all <- length(b_all)


n_events <- sum(my_data$Ninety_day_mortality == 1, na.rm = TRUE)

# 3) Events per variable (EPV)
EPV <- n_events / p_df_all
EPV
```

```
[1] 8.222222
```

```r
## --- Joint hospital block test


idx_h <- grep("^Sjukhus", res$term)   # justera pattern vid behov
if (length(idx_h) > 0) {
  b_h <- as.numeric(comb$estimates[idx_h])
  V_h <- as.matrix(comb$variances[idx_h, idx_h, drop = FALSE])

  if (qr(V_h)$rank < ncol(V_h)) {
    V_h <- V_h + diag(1e-8, nrow(V_h))
  }

  chi2_h <- as.numeric(t(b_h) %*% solve(V_h, b_h))
  df_h   <- length(b_h)
```

```r
  p_h    <- 1 - pchisq(chi2_h, df_h)

  joint_hosp <- data.frame(
    effect = "Hospital block (pooled Wald)",
    chisq  = chi2_h,
    df     = df_h,
    p      = p_h
  )

  write.csv(joint_hosp, "direct_effect_hospital_blocktest.csv", row.names = FALSE)
  print(joint_hosp)
}
```

```
                        effect    chisq df            p
1 Hospital block (pooled Wald) 35.97621  6 2.786135e-06
```

```r
write.csv(
  res,
  file = "direct_effect_model_results.csv",
  row.names = FALSE
)
```
