# Supplementary material for: COVID‐19 Mortality in Swedish Intensive Care Units: A Multicenter Survival Analysis
Source: Acta Anaesthesiol Scand. 2026 Jun 14;70(6):e70279. doi: 10.1111/aas.70279 (PMC13265249; doi:10.1111/aas.70279)
Supplement: Supplementary file 3 — Data S3: Survival analysis and sensitivity testing. [file AAS-70-0-s002.pdf]

Survival analysis and sensitivity testing

Gustaf Forsberg

2026-02-12

Below, relevant packages are loaded and data is imported and prepared for analysis

```
rm(list = ls())

library(pacman)
p_load(readxl, mice, survival, dplyr, lubridate, miceadds, splines, MissMech, naniar,
 ↪  VIM, tidyr, ggplot2, broom, coxme, car, stdReg)
my_data <- read_excel("descriptive_mort_v3.xlsx", sheet = "Blad3")
my_data <- my_data %>%
  mutate(
    Sjukhus = factor(Sjukhus,
                     levels = 1:7,
                     labels = c("Hospital B2", "Hospital C2", "Hospital C1", "Hospital
                      ↪  A1", "Hospital B1", "Hospital B3", "Hospital C3")),
    Sjukvardsregion = factor(Sjukvardsregion,
                             levels = 1:3,
                             labels = c("Region 1", "Region 2", "Region 3")),
    BMI = as.numeric(BMI)
  )


my_data$Woman <- factor(my_data$Woman, levels = c("0", "1"))
my_data$Woman <- relevel(my_data$Woman, ref = "1")
my_data$Current_or_x_smoker <- factor(my_data$Current_or_x_smoker, levels = c("0", "1"))
my_data$Current_or_x_smoker <- relevel(my_data$Current_or_x_smoker, ref = "0")
my_data$Treatment_restr <- factor(my_data$Treatment_restr, levels = c("0", "1"))
my_data$Treatment_restr <- relevel(my_data$Treatment_restr, ref = "0")
```

```
df <- my_data

table(df$Sjukhus)
```

```
Hospital B2 Hospital C2 Hospital C1 Hospital A1 Hospital B1 Hospital B3
        164          88          88         169         139          82
Hospital C3
         17
```

```r
df %>% dplyr::count(Sjukhus)
```

```
# A tibble: 7 x 2
  Sjukhus         n
  <fct>       <int>
1 Hospital B2   164
2 Hospital C2    88
3 Hospital C1    88
4 Hospital A1   169
5 Hospital B1   139
6 Hospital B3    82
7 Hospital C3    17
```

Below, calendar time splines are created and multiple imputation is performed. Then, coxme is performed on each imputed dataset and combined using rubin's rules. Additionally, imputation diagnostics are performed and a complete case analysis is made and exported.

```r
df <- my_data %>%
  mutate(
    admission_date = as.Date(admission_date),
    date_num = as.numeric(admission_date),
    Ninety_day_mortality = as.integer(Ninety_day_mortality),
  )

k1 <- as.numeric(as.Date("2020-07-01"))
k2 <- as.numeric(as.Date("2021-02-16"))

NS <- ns(df$date_num, knots = c(k1, k2))
colnames(NS) <- paste0("cs_date_", seq_len(ncol(NS)))
df <- bind_cols(df, as.data.frame(NS))

vars_keep <- c(
  "Tid_censur_event","Ninety_day_mortality",
  "Current_or_x_smoker","CCI","SAPS3","BMI",
  "Age","Woman","Sjukhus","Sjukvardsregion",
  "admission_date","date_num", colnames(NS)
)

dat <- select(df, any_of(vars_keep))

dat$Current_or_x_smoker <- factor(dat$Current_or_x_smoker, levels = c(0,1), labels =
 ↪  c("No","Yes"))

meth <- make.method(dat); meth[] <- ""
meth["Current_or_x_smoker"] <- "logreg"  # binär
meth["CCI"]                 <- "pmm"     # kvasi-kontinuerlig (alt. polr om ordinal)
meth["SAPS3"]               <- "pmm"
meth["BMI"]                 <- "pmm"

pred <- make.predictorMatrix(dat); pred[,] <- 0

base_preds <- setdiff(vars_keep, c("Current_or_x_smoker","CCI","SAPS3","BMI"))
```

```r
setdiff(c("Tid_censur_event","Ninety_day_mortality","Current_or_x_smoker","CCI","SAPS3","BMI",
          "Age","Woman","Sjukhus","Sjukvardsregion","admission_date","date_num",
          grep("^cs_date_", names(df), value=TRUE)),
        names(dat))
```

```
character(0)
```

```r
pred["Current_or_x_smoker", c(base_preds,"CCI","SAPS3","BMI")] <- 1
pred["CCI",                  c(base_preds,"Current_or_x_smoker","SAPS3","BMI")] <- 1
pred["SAPS3",                c(base_preds,"Current_or_x_smoker","CCI","BMI")] <- 1
pred["BMI",                  c(base_preds,"Current_or_x_smoker","CCI","SAPS3")] <- 1

meth[c("Tid_censur_event","Ninety_day_mortality","Age","Woman",
       "Sjukhus","Sjukvardsregion","admission_date","date_num",
       colnames(NS))] <- ""

m <- 30
set.seed(2025)
imp <- mice(dat, m = m, maxit = 20, method = meth, predictorMatrix = pred, printFlag =
 ↪  FALSE)
```

```
Warning: Number of logged events: 3001
```

```r
# --- Imputationdiagnostics
plot(imp)
```

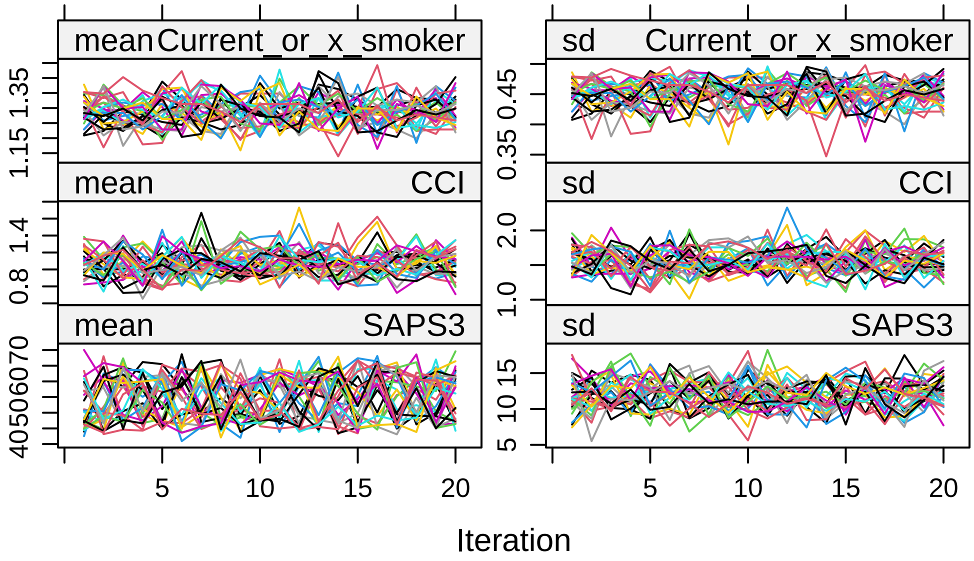

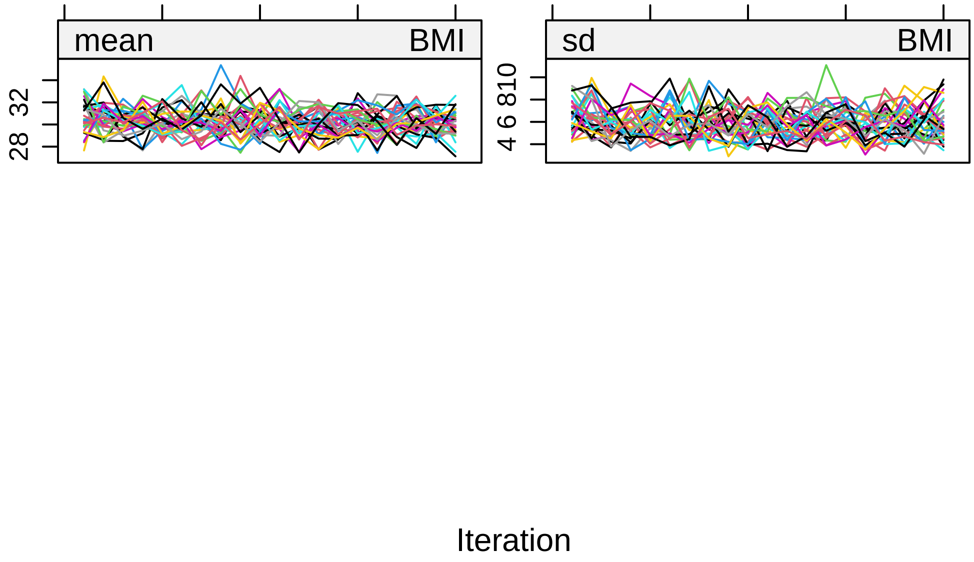


```r
densityplot(imp, ~ BMI + SAPS3 + CCI)
```

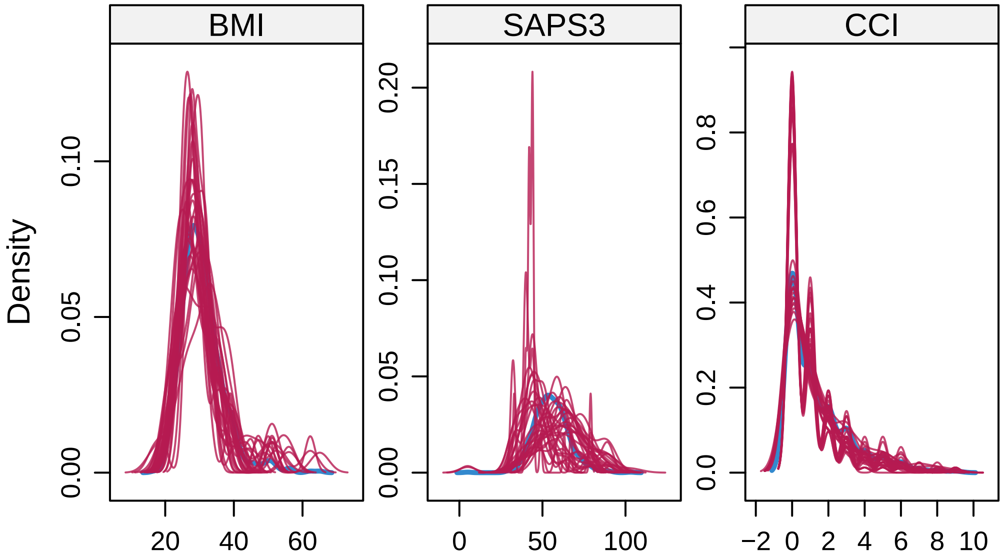


```r
stripplot(imp,  BMI   ~ .imp, jitter = TRUE)
```

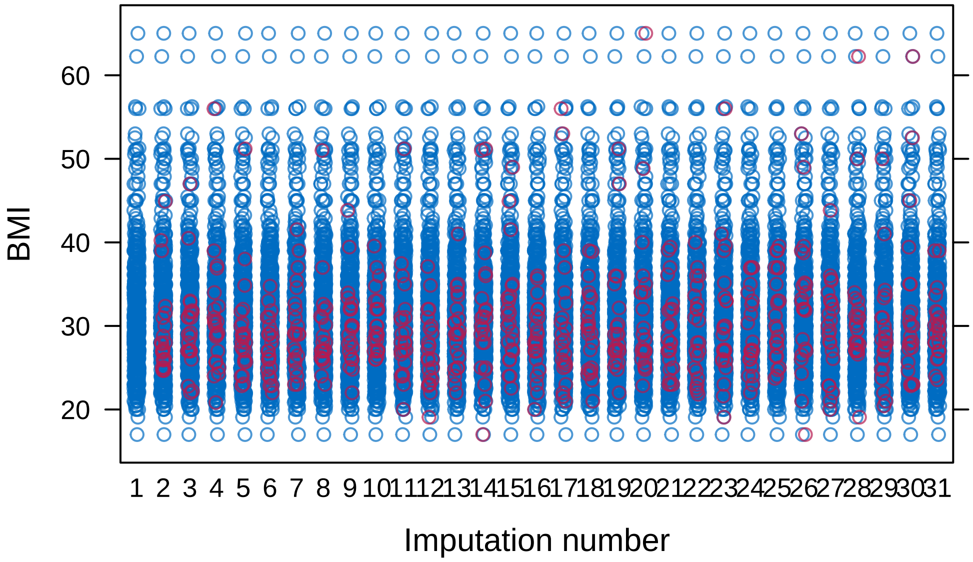


```
stripplot(imp,  SAPS3 ~ .imp, jitter = TRUE)
```

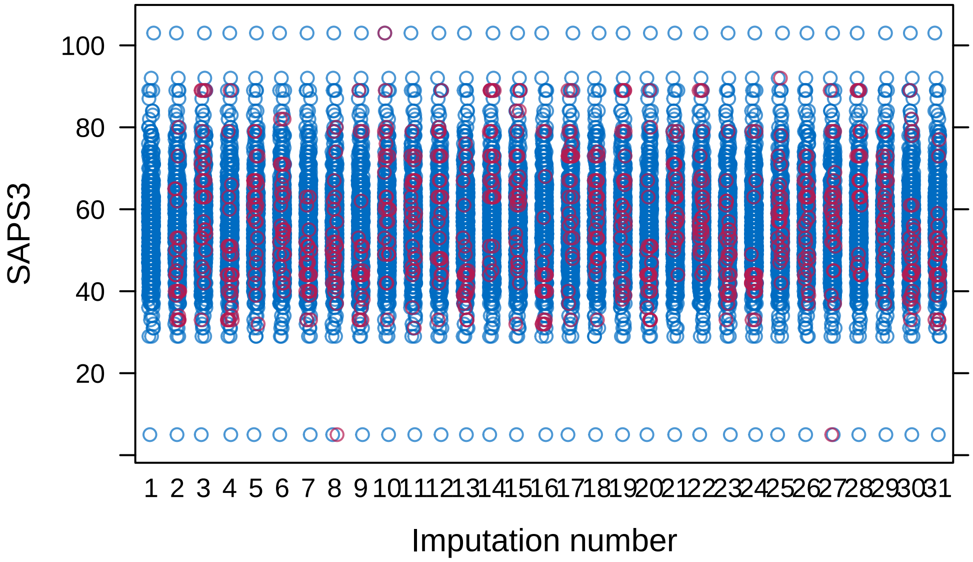


```
stripplot(imp,  CCI   ~ .imp, jitter = TRUE)
```

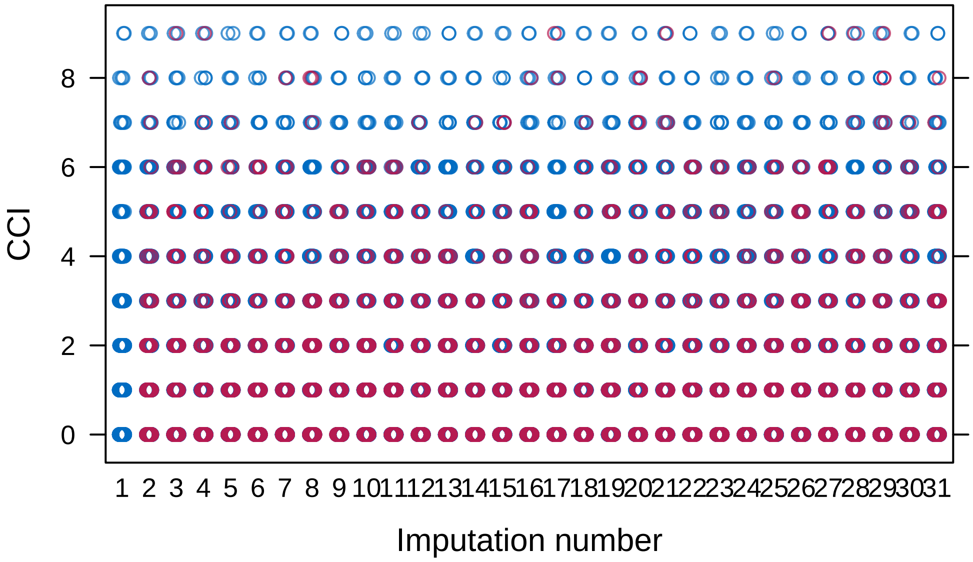


```
# Fraction of Missing Information (FMI)
fitlist_fix <- with(
  imp,
  coxph(
    Surv(Tid_censur_event, Ninety_day_mortality) ~
      Current_or_x_smoker + CCI + SAPS3 + BMI + Age + Woman +
      strata(Sjukhus) +
      strata(Sjukvardsregion) +
      cs_date_1 + cs_date_2 + cs_date_3,
    ties = "breslow", x = TRUE
  )
)
pool_fix <- pool(fitlist_fix)
summ_fix <- summary(pool_fix)

names(summ_fix)
```

```
[1] "term"      "estimate"  "std.error" "statistic" "df"        "p.value"
```

```
want <- c("term","estimate","std.error","fmi","lambda","df","p.value","2.5 %","97.5 %")
summ_fix_out <- summ_fix[, intersect(want, names(summ_fix))]
summ_fix_out
```

```
                   term    estimate   std.error        df      p.value
1 Current_or_x_smokerYes -0.08065577 0.204064402  86.01704 6.936394e-01
2                    CCI  0.01340823 0.053183789 108.63108 8.014314e-01
3                  SAPS3  0.03150094 0.009908474 111.83534 1.910647e-03
4                    BMI -0.01233023 0.017591325 129.21296 4.846090e-01
5                    Age  0.05488063 0.010459779 132.20976 5.992332e-07
6                 Woman0  0.11009890 0.192594876 134.03195 5.685094e-01
```

```
7              cs_date_1 -0.07837764 0.418005987 135.63788 8.515460e-01
8              cs_date_2 -1.61512963 1.669717006 135.70208 3.351112e-01
9              cs_date_3 -0.42734640 0.652748548 130.86324 5.138179e-01
```

```
pf <- pool(fitlist_fix)

names(pf)           # bl.a. "pooled"
```

```
[1] "call"    "m"       "pooled"  "glanced"
```

```
colnames(pf$pooled) # "term","estimate","u","b","t","df","riv","lambda","fmi"
```

```
 [1] "term"     "m"        "estimate" "ubar"     "b"        "t"
 [7] "dfcom"    "df"       "riv"      "lambda"   "fmi"
```

```
tab <- pf$pooled
tab$std.error <- sqrt(tab$t)  # SE = sqrt(total variance)

fmi_tab <- tab[, c("term","estimate","std.error","df","riv","lambda","fmi")]
fmi_tab
```

```
                   term    estimate   std.error        df         riv
1 Current_or_x_smokerYes -0.08065577 0.204064402  86.01704 0.318045675
2                    CCI  0.01340823 0.053183789 108.63108 0.165878357
3                  SAPS3  0.03150094 0.009908474 111.83534 0.147403234
4                    BMI -0.01233023 0.017591325 129.21296 0.049912334
5                    Age  0.05488063 0.010459779 132.20976 0.032006412
6                 Woman0  0.11009890 0.192594876 134.03195 0.020544547
7              cs_date_1 -0.07837764 0.418005987 135.63788 0.009899718
8              cs_date_2 -1.61512963 1.669717006 135.70208 0.009460800
9              cs_date_3 -0.42734640 0.652748548 130.86324 0.040169666
       lambda        fmi
1 0.241300951 0.25834710
2 0.142277585 0.15764467
3 0.128466811 0.14364564
4 0.047539526 0.06194750
5 0.031013772 0.04534685
6 0.020130965 0.03443229
7 0.009802675 0.02408733
8 0.009372132 0.02365639
9 0.038618379 0.05298202
```

```
mean(pf$pooled$fmi, na.rm = TRUE)
```

```
[1] 0.08912109
```

```
# ----  CoxME (random intercept for healthcare county) with splines

est <- list()
vcov_list <- list()
```

```r
for (k in 1:m) {
  d_k <- complete(imp, k)

  fit_k <- coxme::coxme(
    Surv(Tid_censur_event, Ninety_day_mortality) ~
      Current_or_x_smoker + CCI + SAPS3 + BMI + Age + Woman +
      Sjukhus + (1 | Sjukvardsregion) +
      cs_date_1 + cs_date_2 + cs_date_3,
    data = d_k
  )

  b <- stats::coef(fit_k)
  V <- as.matrix(vcov(fit_k))

  est[[k]] <- b
  vcov_list[[k]] <- V
}

pool_rubin <- function(estimates, variances) {
  m <- length(estimates)
  p <- length(estimates[[1]])

  qbar <- Reduce("+", estimates) / m
  ubar <- Reduce("+", variances) / m

  b <- Reduce("+", lapply(estimates, function(q) (q - qbar) %*% t(q - qbar))) / (m - 1)

  tvar <- ubar + (1 + 1/m) * b

  list(estimates = qbar, variances = tvar)
}

comb <- pool_rubin(est, vcov_list)

## --- EPV
n_events_main <- sum(df$Ninety_day_mortality == 1, na.rm = TRUE)


n_par_main <- length(comb$estimates)

# Events per variable (EPV)
EPV_main <- n_events_main / n_par_main
EPV_main
```

```
[1] 9.866667
```

```r
epv_main <- data.frame(
  model        = "Main CoxME 90-day mortality",
  n_events     = n_events_main,
  n_parameters = n_par_main,
  EPV          = EPV_main
)
```

```r
write.csv(epv_main, "main_model_EPV.csv", row.names = FALSE)


res <- data.frame(
  term = names(comb$estimates),
  estimate = comb$estimates,
  se = sqrt(diag(comb$variances))
)
res$HR  <- exp(res$estimate)
res$LCL <- exp(res$estimate - 1.96*res$se)
res$UCL <- exp(res$estimate + 1.96*res$se)
res$p   <- 2*pnorm(-abs(res$estimate/res$se))
res
```

```
                                        term    estimate          se        HR
Current_or_x_smokerYes Current_or_x_smokerYes -0.07383034 0.204959902 0.9288293
CCI                                      CCI  0.01325347 0.053457363 1.0133417
SAPS3                                  SAPS3  0.03167739 0.009953317 1.0321845
BMI                                      BMI -0.01414758 0.017598901 0.9859520
Age                                      Age  0.05522253 0.010461036 1.0567758
Woman0                                Woman0  0.10704297 0.192420028 1.1129821
SjukhusHospital C2         SjukhusHospital C2  1.34453274 0.339308771 3.8363935
SjukhusHospital C1         SjukhusHospital C1  0.86643823 0.368025737 2.3784243
SjukhusHospital A1         SjukhusHospital A1  0.92170505 0.331764863 2.5135725
SjukhusHospital B1         SjukhusHospital B1  1.28608722 0.322893992 3.6186000
SjukhusHospital B3         SjukhusHospital B3  1.62076130 0.347904584 5.0569387
SjukhusHospital C3         SjukhusHospital C3  1.55562644 0.578069893 4.7380537
cs_date_1                          cs_date_1 -0.07814059 0.419331642 0.9248344
cs_date_2                          cs_date_2 -1.59386700 1.673590627 0.2031386
cs_date_3                          cs_date_3 -0.44555275 0.653551496 0.6404702
                               LCL       UCL            p
Current_or_x_smokerYes 0.621542027  1.388038 7.186838e-01
CCI                    0.912540362  1.125278 8.041917e-01
SAPS3                  1.012243227  1.052519 1.459612e-03
BMI                    0.952522615  1.020555 4.214604e-01
Age                    1.035328636  1.078667 1.299774e-07
Woman0                 0.763302940  1.622854 5.780068e-01
SjukhusHospital C2     1.972866471  7.460168 7.414911e-05
SjukhusHospital C1     1.156164179  4.892819 1.855827e-02
SjukhusHospital A1     1.311859925  4.816099 5.466304e-03
SjukhusHospital B1     1.921709192  6.813865 6.805036e-05
SjukhusHospital B3     2.557085954 10.000692 3.183094e-06
SjukhusHospital C3     1.525938220 14.711705 7.122329e-03
cs_date_1              0.406557215  2.103809 8.521738e-01
cs_date_2              0.007641934  5.399847 3.409126e-01
cs_date_3              0.177903558  2.305755 4.954028e-01
```

```r
N_me <- integer(m)
E_me <- integer(m)

for (k in 1:m) {
  d_k <- complete(imp, k)
```

```r
  N_me[k] <- nrow(d_k)
  E_me[k] <- sum(d_k$Ninety_day_mortality == 1, na.rm = TRUE)
}

cat("coxme - n per imputat:\n");      print(table(N_me))
```

```
coxme - n per imputat:

N_me
747
 30
```

```r
cat("coxme - events per imputat:\n"); print(table(E_me))
```

```
coxme - events per imputat:

E_me
148
 30
```

```r
# --- Complete-case
needed <- c("Tid_censur_event","Ninety_day_mortality",
            "Current_or_x_smoker","CCI","SAPS3","BMI",
            "Age","Woman","Sjukhus","Sjukvardsregion",
            "cs_date_1","cs_date_2","cs_date_3")

cc <- dat[, intersect(needed, names(dat))]

num_safe <- function(x) if (is.factor(x)) as.numeric(as.character(x)) else as.numeric(x)
for (v in c("BMI","CCI","SAPS3","Age")) if (v %in% names(cc)) cc[[v]] <-
↪  num_safe(cc[[v]])

if ("Sjukhus" %in% names(cc))         cc$Sjukhus         <- factor(cc$Sjukhus,
↪  levels = levels(dat$Sjukhus))
if ("Sjukvardsregion" %in% names(cc)) cc$Sjukvardsregion <- factor(cc$Sjukvardsregion,
↪  levels = levels(dat$Sjukvardsregion))
if ("Current_or_x_smoker" %in% names(cc)) {
  cc$Current_or_x_smoker <- factor(cc$Current_or_x_smoker, levels =
↪  levels(dat$Current_or_x_smoker))
  cc$Current_or_x_smoker <- relevel(cc$Current_or_x_smoker, ref = "No")  # samma ref som
↪  MI
}
if ("Woman" %in% names(cc)) {
  cc$Woman <- factor(cc$Woman, levels = levels(dat$Woman))
  if ("1" %in% levels(cc$Woman)) cc$Woman <- relevel(cc$Woman, ref = "1")
}

print(colSums(is.na(cc)))
```

```
    Tid_censur_event Ninety_day_mortality  Current_or_x_smoker
                   0                    0                  201
                 CCI                SAPS3                  BMI
                 130                   28                   23
                 Age                Woman              Sjukhus
                   0                    0                    0
     Sjukvardsregion              cs_date_1            cs_date_2
                   0                    0                    0
           cs_date_3
                   0
```

```
cc <- cc[complete.cases(cc), ]
print(nrow(cc))  # ska nu vara > 0
```

```
[1] 401
```

```
fit_cc <- coxme::coxme(
  Surv(Tid_censur_event, Ninety_day_mortality) ~
    Current_or_x_smoker + CCI + SAPS3 + BMI + Age + Woman +
    Sjukhus + (1 | Sjukvardsregion) +
    cs_date_1 + cs_date_2 + cs_date_3,
  data = cc
)
summary(fit_cc)
```

```
Mixed effects coxme model
 Formula: Surv(Tid_censur_event, Ninety_day_mortality) ~ Current_or_x_smoker +      CCI + SAPS3 + BMI +
    Data: cc

  events, n = 91, 401

Random effects:
            group  variable          sd     variance
1 Sjukvardsregion Intercept 0.008966398 8.039629e-05
                  Chisq df          p   AIC   BIC
Integrated loglik 87.24 16 8.044e-12 55.24 15.07
 Penalized loglik 87.24 15 3.232e-12 57.24 19.58

Fixed effects:
                            coef exp(coef)  se(coef)     z        p
Current_or_x_smokerYes -0.114755  0.891585  0.226440 -0.51 0.612310
CCI                    -0.036169  0.964478  0.066649 -0.54 0.587357
SAPS3                   0.044845  1.045866  0.012791  3.51 0.000455
BMI                    -0.006101  0.993917  0.023775 -0.26 0.797469
Age                     0.042463  1.043377  0.013624  3.12 0.001828
Woman0                  0.003174  1.003179  0.237229  0.01 0.989327
SjukhusHospital C2      1.581658  4.863010  0.817276  1.94 0.052956
SjukhusHospital C1      1.676545  5.347052  0.787321  2.13 0.033219
SjukhusHospital A1      1.466038  4.332039  0.755428  1.94 0.052298
SjukhusHospital B1      1.966779  7.147618  0.748843  2.63 0.008629
SjukhusHospital B3      2.702409 14.915620  0.768051  3.52 0.000434
SjukhusHospital C3      2.890020 17.993668  0.881589  3.28 0.001045
```

```
cs_date_1              0.571214  1.770415  0.652803  0.88 0.381564
cs_date_2             -3.226266  0.039705  2.456006 -1.31 0.188973
cs_date_3             -1.031341  0.356528  1.017151 -1.01 0.310606
```

```
coef_cc <- stats::coef(fit_cc)
V_cc    <- as.matrix(vcov(fit_cc))

cc_fix <- data.frame(
  term     = names(coef_cc),
  estimate = as.numeric(coef_cc),
  se       = sqrt(diag(V_cc))
)
cc_fix$HR  <- exp(cc_fix$estimate)
cc_fix$LCL <- exp(cc_fix$estimate - 1.96*cc_fix$se)
cc_fix$UCL <- exp(cc_fix$estimate + 1.96*cc_fix$se)
cc_fix$p   <- 2*pnorm(-abs(cc_fix$estimate / cc_fix$se))


cc_res <- data.frame(
  term     = names(fixef(fit_cc)),
  estimate = fixef(fit_cc),
  se       = sqrt(diag(vcov(fit_cc)))
)

cc_res$HR  <- exp(cc_res$estimate)
cc_res$LCL <- exp(cc_res$estimate - 1.96 * cc_res$se)
cc_res$UCL <- exp(cc_res$estimate + 1.96 * cc_res$se)
cc_res$p   <- 2 * pnorm(-abs(cc_res$estimate / cc_res$se))

print(cc_res)
```

```
                                       term     estimate         se
Current_or_x_smokerYes Current_or_x_smokerYes -0.114754909 0.22644007
CCI                                         CCI -0.036168521 0.06664913
SAPS3                                     SAPS3  0.044845093 0.01279075
BMI                                         BMI -0.006101168 0.02377471
Age                                         Age  0.042462885 0.01362370
Woman0                                   Woman0  0.003173556 0.23722895
SjukhusHospital C2          SjukhusHospital C2  1.581657591 0.81727625
SjukhusHospital C1          SjukhusHospital C1  1.676545425 0.78732087
SjukhusHospital A1          SjukhusHospital A1  1.466038329 0.75542820
SjukhusHospital B1          SjukhusHospital B1  1.966779164 0.74884310
SjukhusHospital B3          SjukhusHospital B3  2.702408982 0.76805120
SjukhusHospital C3          SjukhusHospital C3  2.890019908 0.88158936
cs_date_1                             cs_date_1  0.571213731 0.65280261
cs_date_2                             cs_date_2 -3.226265863 2.45600585
cs_date_3                             cs_date_3 -1.031341330 1.01715051
                               HR          LCL        UCL            p
Current_or_x_smokerYes  0.89158464 0.5720222125   1.389672 0.6123104048
CCI                     0.96447775 0.8463681828   1.099069 0.5873566286
SAPS3                   1.04586584 1.0199720530   1.072417 0.0004547974
BMI                     0.99391741 0.9486649412   1.041328 0.7974688564
Age                     1.04337733 1.0158852877   1.071613 0.0018280083
Woman0                  1.00317860 0.6301513943   1.597025 0.9893265326
```

```
SjukhusHospital C2      4.86301002 0.9799988635  24.131524 0.0529560650
SjukhusHospital C1      5.34705223 1.1427030196  25.020471 0.0332186217
SjukhusHospital A1      4.33203899 0.9855051326  19.042581 0.0522980792
SjukhusHospital B1      7.14761807 1.6471502645  31.016262 0.0086287372
SjukhusHospital B3     14.91561993 3.3102662824  67.207801 0.0004339494
SjukhusHospital C3     17.99366782 3.1966543804 101.284669 0.0010447401
cs_date_1               1.77041455 0.4924908591   6.364316 0.3815643624
cs_date_2               0.03970549 0.0003222969   4.891532 0.1889730922
cs_date_3               0.35652842 0.0485599418   2.617641 0.3106058746
```

Below, an identical analysis to the primary model but excluding all transferred patients is performed.

```r
est <- vector("list", m)
vcov_list <- vector("list", m)

pool_rubin <- function(estimates, variances) {
  m <- length(estimates)
  p <- length(estimates[[1]])


  qbar <- Reduce("+", estimates) / m


  ubar <- Reduce("+", variances) / m


  b <- Reduce(
    "+",
    lapply(estimates, function(q) {
      d <- q - qbar
      d %*% t(d)
    })
  ) / (m - 1)


  tvar <- ubar + (1 + 1 / m) * b

  list(
    estimates = qbar,
    variances = tvar
  )
}

for (k in 1:m) {
  d_k <- complete(imp, k)


  d_k$id <- my_data$id


  d_k$Transfer_within_hospital_region <-
    my_data$Transfer_within_hospital_region[ match(d_k$id, my_data$id) ]
```

```r
  d_k <- subset(d_k,
                !is.na(Transfer_within_hospital_region) &
                  Transfer_within_hospital_region == 0)

  fit_k <- coxme::coxme(
    Surv(Tid_censur_event, Ninety_day_mortality) ~
      Current_or_x_smoker + CCI + SAPS3 + BMI + Age + Woman +
      Sjukhus + (1 | Sjukvardsregion) +
      cs_date_1 + cs_date_2 + cs_date_3,
    data = d_k
  )

  est[[k]] <- stats::coef(fit_k)
  vcov_list[[k]] <- as.matrix(vcov(fit_k))
}

comb <- pool_rubin(est, vcov_list)

stopifnot(!anyNA(match(my_data$id, my_data$id)))  # trivial
stopifnot(!anyNA(match(d_k$id, my_data$id)))      # kör efter att du skapat d_k


beta <- comb$estimates


V <- comb$variances


se <- sqrt(diag(V))


lcl <- beta - 1.96 * se
ucl <- beta + 1.96 * se


res <- data.frame(
  term = names(beta),
  HR   = exp(beta),
  LCL  = exp(lcl),
  UCL  = exp(ucl),
  row.names = NULL
)

res$HR_CI <- sprintf(
  "%.2f (%.2f-%.2f)",
  res$HR, res$LCL, res$UCL
)

write.csv(res, "coxme_MI_no_transfers.csv", row.names = FALSE)


nrow(res)
```

```
[1] 15
```

```r
stopifnot(!anyNA(res))


summary(res$HR)
```

```
   Min. 1st Qu.  Median    Mean 3rd Qu.    Max. 
 0.0831  1.0041  1.0615  1.9844  3.0489  5.5696 
```

```r
epv_df <- data.frame(k = 1:m, events = NA_integer_, p = NA_integer_, epv = NA_real_)

for (k in 1:m) {
  d_k <- complete(imp, k)

  d_k$id <- my_data$id
  d_k$Transfer_within_hospital_region <-
    my_data$Transfer_within_hospital_region[ match(d_k$id, my_data$id) ]

  d_k <- subset(d_k, !is.na(Transfer_within_hospital_region) &
                     Transfer_within_hospital_region == 0)


  fit_k <- coxme::coxme(
    Surv(Tid_censur_event, Ninety_day_mortality) ~
      Current_or_x_smoker + CCI + SAPS3 + BMI + Age + Woman +
      Sjukhus + (1 | Sjukvardsregion) +
      cs_date_1 + cs_date_2 + cs_date_3,
    data = d_k
  )

  events_k <- sum(d_k$Ninety_day_mortality == 1, na.rm = TRUE)
  p_k      <- length(stats::coef(fit_k))   # antal fixed-effect parametrar

  epv_df[epv_df$k == k, c("events","p","epv")] <- c(events_k, p_k, events_k / p_k)
}


summary_epv <- with(epv_df, c(
  events_mean = mean(events),
  events_min  = min(events),
  events_max  = max(events),
  p           = unique(p),
  epv_mean    = mean(epv),
  epv_median  = median(epv),
  epv_min     = min(epv),
  epv_max     = max(epv)
))

epv_df
```

```
    k events  p      epv
1   1    124 15 8.266667
2   2    124 15 8.266667
3   3    124 15 8.266667
```

```
4   4     124 15 8.266667
5   5     124 15 8.266667
6   6     124 15 8.266667
7   7     124 15 8.266667
8   8     124 15 8.266667
9   9     124 15 8.266667
10 10     124 15 8.266667
11 11     124 15 8.266667
12 12     124 15 8.266667
13 13     124 15 8.266667
14 14     124 15 8.266667
15 15     124 15 8.266667
16 16     124 15 8.266667
17 17     124 15 8.266667
18 18     124 15 8.266667
19 19     124 15 8.266667
20 20     124 15 8.266667
21 21     124 15 8.266667
22 22     124 15 8.266667
23 23     124 15 8.266667
24 24     124 15 8.266667
25 25     124 15 8.266667
26 26     124 15 8.266667
27 27     124 15 8.266667
28 28     124 15 8.266667
29 29     124 15 8.266667
30 30     124 15 8.266667
```

```
summary_epv
```

```
events_mean  events_min  events_max           p    epv_mean  epv_median
 124.000000  124.000000  124.000000   15.000000    8.266667    8.266667
    epv_min     epv_max
   8.266667    8.266667
```

Below, Coxme variance is extracted and model diagnostics are performed

```
library(coxme)

est <- list()
vcov_list <- list()
re_sd_region <- numeric(m)

for (k in 1:m) {
  d_k <- complete(imp, k)

  fit_k <- coxme::coxme(
    Surv(Tid_censur_event, Ninety_day_mortality) ~
      Current_or_x_smoker + CCI + SAPS3 + BMI + Age + Woman +
      cs_date_1 + cs_date_2 + cs_date_3 + Sjukhus +
      (1 | Sjukvardsregion),
    data = d_k
  )
```

```r
  b <- stats::coef(fit_k)
  V <- as.matrix(vcov(fit_k))
  est[[k]] <- b
  vcov_list[[k]] <- V

  vc <- try(VarCorr(fit_k), silent = TRUE)
  if (!inherits(vc, "try-error")) {
    re_sd_region[k] <- sqrt(as.numeric(vc))
  } else {
    sm <- capture.output(print(fit_k))
    mtxt <- grep("Sjukvardsregion", sm, value = TRUE)
    re_sd_region[k] <- suppressWarnings(as.numeric(sub(".*=\\s*([0-9\\.eE+-]+).*", "\\1",
 ↪  mtxt)))
  }
}

pool_rubin <- function(estimates, variances){
  m <- length(estimates)
  qbar <- Reduce("+", estimates)/m
  ubar <- Reduce("+", variances)/m
  b <- Reduce("+", lapply(estimates, function(q) (q - qbar) %*% t(q - qbar))) / (m - 1)
  tvar <- ubar + (1 + 1/m)*b
  list(estimates = qbar, variances = tvar)
}
comb <- pool_rubin(est, vcov_list)

res_fix <- data.frame(
  term = names(comb$estimates),
  estimate = comb$estimates,
  se = sqrt(diag(comb$variances))
)
res_fix$HR  <- exp(res_fix$estimate)
res_fix$LCL <- exp(res_fix$estimate - 1.96*res_fix$se)
res_fix$UCL <- exp(res_fix$estimate + 1.96*res_fix$se)
res_fix$p   <- 2*pnorm(-abs(res_fix$estimate/res_fix$se))

cc <- na.omit(dat)
fit_cc <- coxme::coxme(Surv(Tid_censur_event, Ninety_day_mortality) ~
                        Current_or_x_smoker + CCI + SAPS3 + BMI + Age + Woman +
                        cs_date_1 + cs_date_2 + cs_date_3 + (1 | Sjukvardsregion),
                       data = cc)
summary(fit_cc)
```

```
Mixed effects coxme model
 Formula: Surv(Tid_censur_event, Ninety_day_mortality) ~ Current_or_x_smoker +      CCI + SAPS3 + BMI +
    Data: cc

  events, n = 91, 401

Random effects:
            group  variable        sd  variance
1 Sjukvardsregion Intercept 0.7073471 0.5003399
                  Chisq    df         p   AIC   BIC
```

```
Integrated loglik 69.82 10.00 4.799e-11 49.82 24.71
 Penalized loglik 76.85 10.82 4.945e-12 55.22 28.05

Fixed effects:
                          coef exp(coef)  se(coef)     z        p
Current_or_x_smokerYes -0.121048  0.885992  0.223577 -0.54 0.588223
CCI                    -0.055425  0.946083  0.065971 -0.84 0.400827
SAPS3                   0.049576  1.050825  0.012333  4.02 5.83e-05
BMI                    -0.002435  0.997568  0.022964 -0.11 0.915566
Age                     0.043783  1.044756  0.013218  3.31 0.000925
Woman0                  0.075151  1.078047  0.235396  0.32 0.749535
cs_date_1               0.621269  1.861288  0.631251  0.98 0.325024
cs_date_2              -3.801841  0.022330  2.445891 -1.55 0.120094
cs_date_3              -1.226411  0.293343  0.973412 -1.26 0.207702
```

```r
re_summary <- data.frame(
  metric = c("region RE SD - mean","region RE SD - median","region RE SD -
   ↪  IQR_low","region RE SD - IQR_high"),
  value  = c(mean(re_sd_region, na.rm=TRUE),
             median(re_sd_region, na.rm=TRUE),
             quantile(re_sd_region, .25, na.rm=TRUE),
             quantile(re_sd_region, .75, na.rm=TRUE))
)


### VIF

d_vif <- complete(imp, 1)

set.seed(1)
d_vif$y_dummy <- rnorm(nrow(d_vif))

form_vif <- y_dummy ~ Current_or_x_smoker + CCI + SAPS3 + BMI + Age + Woman + Sjukhus +
  cs_date_1 + cs_date_2 + cs_date_3

fit_vif <- lm(form_vif, data = d_vif)

vift <- car::vif(fit_vif)
vif_tab <- as.data.frame(vift)

if ("Df" %in% colnames(vif_tab)) {
  vif_tab$GVIF_adj <- vif_tab$GVIF^(1/(2*vif_tab$Df))
}
vif_tab
```

```
                        GVIF Df GVIF^(1/(2*Df)) GVIF_adj
Current_or_x_smoker 1.126992  1        1.061599 1.061599
CCI                 1.243662  1        1.115196 1.115196
SAPS3               1.543648  1        1.242436 1.242436
BMI                 1.114537  1        1.055716 1.055716
Age                 1.643050  1        1.281815 1.281815
Woman               1.030666  1        1.015217 1.015217
Sjukhus             1.199640  6        1.015284 1.015284
cs_date_1           1.416081  1        1.189992 1.189992
```

```
cs_date_2           1.422188  1        1.192555 1.192555
cs_date_3           1.801542  1        1.342215 1.342215
```

```
d_ph <- complete(imp, 1)

fit_ph <- coxph(
  Surv(Tid_censur_event, Ninety_day_mortality) ~
    Current_or_x_smoker + CCI + SAPS3 + BMI + Age + Woman + Sjukhus +
    cs_date_1 + cs_date_2 + cs_date_3 +
    strata(Sjukvardsregion),
  data = d_ph,
  x = TRUE
)
```

```
Warning in coxph.fit(X, Y, istrat, offset, init, control, weights = weights, :
Loglik converged before variable 9,10 ; coefficient may be infinite.
```

```
ph_test <- cox.zph(fit_ph, transform = "km")
ph_test
```

```
                      chisq df     p
Current_or_x_smoker  0.4036  1 0.525
CCI                  0.0551  1 0.814
SAPS3                0.3398  1 0.560
BMI                  0.3357  1 0.562
Age                  1.7823  1 0.182
Woman                5.0471  1 0.025
Sjukhus              3.8662  5 0.569
cs_date_1            5.1598  1 0.023
cs_date_2            5.9027  1 0.015
cs_date_3            0.0135  1 0.908
GLOBAL              23.3422 14 0.055
```

Below, g-standardization of 90-day mortality is calculated

```
form_cox <- as.formula(
  Surv(Tid_censur_event, Ninety_day_mortality) ~
    Current_or_x_smoker + CCI + SAPS3 + BMI + Age + Woman +
    Sjukhus + cs_date_1 + cs_date_2 + cs_date_3
)

m_sub <- 10
risk_list <- list()
se_list   <- list()
hosp_names <- NULL

for (k in 1:m_sub) {
  d_k <- complete(imp, k)

  fit_k <- coxph(form_cox, data = d_k, ties = "breslow")
  std_k <- stdCoxph(fit = fit_k, data = d_k, X = "Sjukhus")
```

```
  s <- summary(std_k, se = TRUE)
  t_index <- which.min(abs(s$tsum - 90))

  tab <- s$est.table[[t_index]]
  tab <- as.data.frame(tab, stringsAsFactors = FALSE)

  cn <- tolower(colnames(tab))
  pick <- function(choices){ ix <- match(choices, cn); ix[!is.na(ix)][1] }
  i_surv <- pick(c("est","surv","survival","std.surv"))
  i_lcl  <- pick(c("lower","lcl","ci.low","ci_lower"))
  i_ucl  <- pick(c("upper","ucl","ci.high","ci_upper"))
  i_se   <- pick(c("se","std.err","stderr"))

  S    <- as.numeric(tab[[ if (!is.na(i_surv)) i_surv else 1 ]])
  risk <- 1 - S

  if (!is.na(i_se)) {
    se_risk <- as.numeric(tab[[ i_se ]])
    se_risk <- se_risk
  } else if (!is.na(i_lcl) && !is.na(i_ucl)) {
    S_lo <- as.numeric(tab[[ i_lcl ]])
    S_hi <- as.numeric(tab[[ i_ucl ]])
    se_S <- (S_hi - S_lo) / 3.92
    se_risk <- se_S
  } else {
    se_risk <- rep(NA_real_, length(risk))
  }

  risk_list[[k]] <- risk
  se_list[[k]]   <- se_risk

  if (is.null(hosp_names)) {
    if (!is.null(rownames(tab)) && length(rownames(tab))==length(risk)) {
      hosp_names <- rownames(tab)
    } else {
      hosp_names <- levels(factor(d_k$Sjukhus))
      if (length(hosp_names) != length(risk)) hosp_names <- paste0("Hospital_",
       ↪  seq_along(risk))
    }
  }
}


risk_mat <- do.call(cbind, risk_list)
se_mat   <- do.call(cbind, se_list)

qbar <- rowMeans(risk_mat, na.rm = TRUE)
ubar <- rowMeans(se_mat^2, na.rm = TRUE)
ubar[!is.finite(ubar)] <- 0

bvar <- apply(risk_mat, 1, var, na.rm = TRUE)

tvar <- ubar + (1 + 1/m_sub) * bvar
se_p <- sqrt(tvar)
```

```r
LCL <- pmax(0, qbar - 1.96 * se_p)
UCL <- pmin(1, qbar + 1.96 * se_p)

std_tab <- data.frame(
  Sjukhus    = hosp_names,
  time       = 90,
  Mortality = qbar,
  LCL        = LCL,
  UCL        = UCL,
  row.names = NULL
)

std_tab[order(-std_tab$Mortality), ]
```

```
      Sjukhus time  Mortality        LCL        UCL
6 Hospital B3   90 0.31810527 0.30962385 0.32658670
7 Hospital C3   90 0.30448390 0.29361392 0.31535387
2 Hospital C2   90 0.25813127 0.25042032 0.26584222
5 Hospital B1   90 0.24866836 0.24044411 0.25689261
4 Hospital A1   90 0.18640688 0.18205212 0.19076165
3 Hospital C1   90 0.17657484 0.17015477 0.18299491
1 Hospital B2   90 0.08344381 0.08145727 0.08543036
```

```r
write.csv(std_tab, "standardiserad_mortalitet_MI_pooled.csv", row.names = FALSE)
```
