# Supplementary material for: COVID‐19 Mortality in Swedish Intensive Care Units: A Multicenter Survival Analysis
Source: Acta Anaesthesiol Scand. 2026 Jun 14;70(6):e70279. doi: 10.1111/aas.70279 (PMC13265249; doi:10.1111/aas.70279)
Supplement: Supplementary file 4 — Data S4: Treatment restrictions. [file AAS-70-0-s010.pdf]

Treatment restrictions

Gustaf Forsberg

2026-05-15

Treatment restriction

Complementary analysis exploring treatment restrictions.

```
rm(list = ls())

library(pacman)
p_load(readxl, mice, survival, dplyr, lubridate, miceadds, splines, MissMech, naniar,
 ↪  VIM, tidyr, ggplot2, broom, coxme, car, stdReg)
my_data <- read_excel("descriptive_mort_v3.xlsx", sheet = "Blad3")
my_data <- my_data %>%
  mutate(
    Sjukhus = factor(Sjukhus,
                     levels = 1:7,
                     labels = c("Hospital B2", "Hospital C2", "Hospital C1", "Hospital
                      ↪  A1", "Hospital B1", "Hospital B3", "Hospital C3")),
    Sjukvardsregion = factor(Sjukvardsregion,
                             levels = 1:3,
                             labels = c("Region 1", "Region 2", "Region 3")),
    BMI = as.numeric(BMI)
  )


my_data$Woman <- factor(my_data$Woman, levels = c("0", "1"))
my_data$Woman <- relevel(my_data$Woman, ref = "1")
my_data$Current_or_x_smoker <- factor(my_data$Current_or_x_smoker, levels = c("0", "1"))
my_data$Current_or_x_smoker <- relevel(my_data$Current_or_x_smoker, ref = "0")
my_data$Treatment_restr <- factor(my_data$Treatment_restr, levels = c("0", "1"))
my_data$Treatment_restr <- relevel(my_data$Treatment_restr, ref = "0")


df <- my_data

table(df$Sjukhus)
```

```
Hospital B2 Hospital C2 Hospital C1 Hospital A1 Hospital B1 Hospital B3
        164          88          88         169         139          82
Hospital C3
         17
```

```r
df %>% dplyr::count(Sjukhus)
```

```
# A tibble: 7 x 2
  Sjukhus         n
  <fct>       <int>
1 Hospital B2   164
2 Hospital C2    88
3 Hospital C1    88
4 Hospital A1   169
5 Hospital B1   139
6 Hospital B3    82
7 Hospital C3    17
```

Below, a mice imputation is made and the same analysis as for the primary model is run but including treatment restrictions. As this variable may influence causal pathways both upstream and downstream to the exposure variable of interest (hospital), the analysis should primarily be interpreted as exploratory and hypothesis generating.

Additionally, a model excluding all patients with treatment restrictions is run as a complementary sensetivity analysis.

```r
library(dplyr)
library(splines)
library(mice)

df <- my_data %>%
  mutate(
    admission_date = as.Date(admission_date),
    date_num = as.numeric(admission_date),
    Ninety_day_mortality = as.integer(Ninety_day_mortality),
    Treatment_restr = as.integer(Treatment_restr)
  )

k1 <- as.numeric(as.Date("2020-07-01"))
k2 <- as.numeric(as.Date("2021-02-16"))

NS <- ns(df$date_num, knots = c(k1, k2))
colnames(NS) <- paste0("cs_date_", seq_len(ncol(NS)))
df <- bind_cols(df, as.data.frame(NS))

vars_keep <- c(
  "Tid_censur_event","Ninety_day_mortality",
  "Current_or_x_smoker","CCI","SAPS3","BMI",
  "Age","Woman","Sjukhus","Sjukvardsregion",
  "Treatment_restr",
  "admission_date","date_num", colnames(NS)
)

dat <- select(df, any_of(vars_keep))

dat$Current_or_x_smoker <- factor(
  dat$Current_or_x_smoker,
  levels = c(0, 1),
  labels = c("No", "Yes")
)
```

```r
meth <- make.method(dat)
meth[] <- ""

meth["Current_or_x_smoker"] <- "logreg"
meth["CCI"]                 <- "pmm"
meth["SAPS3"]               <- "pmm"
meth["BMI"]                 <- "pmm"

pred <- make.predictorMatrix(dat)
pred[,] <- 0

base_preds <- setdiff(
  vars_keep,
  c("Current_or_x_smoker", "CCI", "SAPS3", "BMI")
)

setdiff(
  c("Tid_censur_event","Ninety_day_mortality","Current_or_x_smoker","CCI","SAPS3","BMI",
    "Age","Woman","Sjukhus","Sjukvardsregion","Treatment_restr",
    "admission_date","date_num",
    grep("^cs_date_", names(df), value = TRUE)),
  names(dat)
)
```

```
character(0)
```

```r
pred["Current_or_x_smoker", c(base_preds, "CCI", "SAPS3", "BMI")] <- 1
pred["CCI",                 c(base_preds, "Current_or_x_smoker", "SAPS3", "BMI")] <- 1
pred["SAPS3",               c(base_preds, "Current_or_x_smoker", "CCI", "BMI")] <- 1
pred["BMI",                 c(base_preds, "Current_or_x_smoker", "CCI", "SAPS3")] <- 1

meth[c("Tid_censur_event","Ninety_day_mortality","Age","Woman",
       "Sjukhus","Sjukvardsregion","Treatment_restr",
       "admission_date","date_num",
       colnames(NS))] <- ""

m <- 30
set.seed(2025)

imp <- mice(
  dat,
  m = m,
  maxit = 20,
  method = meth,
  predictorMatrix = pred,
  printFlag = FALSE
)
```

```
Warning: Number of logged events: 3001
```

```r
# ----  CoxME (random intercept for healthcare county) with splines

est_adj <- list()
vcov_adj <- list()

N_adj <- integer(m)
E_adj <- integer(m)

for (k in 1:m) {
  d_k <- complete(imp, k)

  fit_k <- coxme::coxme(
    Surv(Tid_censur_event, Ninety_day_mortality) ~
      Current_or_x_smoker + CCI + SAPS3 + BMI + Age + Woman +
      Treatment_restr +
      Sjukhus + (1 | Sjukvardsregion) +
      cs_date_1 + cs_date_2 + cs_date_3,
    data = d_k
  )

  b <- stats::coef(fit_k)
  V <- as.matrix(vcov(fit_k))

  est_adj[[k]] <- b
  vcov_adj[[k]] <- V

  N_adj[k] <- nrow(d_k)
  E_adj[k] <- sum(d_k$Ninety_day_mortality == 1, na.rm = TRUE)
}

pool_rubin <- function(estimates, variances) {
  m <- length(estimates)
  p <- length(estimates[[1]])

  qbar <- Reduce("+", estimates) / m
  ubar <- Reduce("+", variances) / m

  b <- Reduce("+", lapply(estimates, function(q) (q - qbar) %*% t(q - qbar))) / (m - 1)

  tvar <- ubar + (1 + 1/m) * b

  list(estimates = qbar, variances = tvar)
}

comb_adj <- pool_rubin(est_adj, vcov_adj)

res_adj <- data.frame(
  model = "Additionally adjusted for treatment restriction",
  term = names(comb_adj$estimates),
  estimate = as.numeric(comb_adj$estimates),
  se = sqrt(diag(comb_adj$variances))
)

res_adj$HR  <- exp(res_adj$estimate)
```

```r
res_adj$LCL <- exp(res_adj$estimate - 1.96 * res_adj$se)
res_adj$UCL <- exp(res_adj$estimate + 1.96 * res_adj$se)
res_adj$p   <- 2 * pnorm(-abs(res_adj$estimate / res_adj$se))

res_adj$n_mean <- mean(N_adj)
res_adj$events_mean <- mean(E_adj)
res_adj$n_parameters <- length(comb_adj$estimates)
res_adj$EPV <- mean(E_adj) / length(comb_adj$estimates)

write.csv(
  res_adj,
  "coxme_adjusted_for_treatment_restriction.csv",
  row.names = FALSE
)

res_adj
```

```
                                                                 model
Current_or_x_smokerYes Additionally adjusted for treatment restriction
CCI                    Additionally adjusted for treatment restriction
SAPS3                  Additionally adjusted for treatment restriction
BMI                    Additionally adjusted for treatment restriction
Age                    Additionally adjusted for treatment restriction
Woman0                 Additionally adjusted for treatment restriction
Treatment_restr        Additionally adjusted for treatment restriction
SjukhusHospital C2     Additionally adjusted for treatment restriction
SjukhusHospital C1     Additionally adjusted for treatment restriction
SjukhusHospital A1     Additionally adjusted for treatment restriction
SjukhusHospital B1     Additionally adjusted for treatment restriction
SjukhusHospital B3     Additionally adjusted for treatment restriction
SjukhusHospital C3     Additionally adjusted for treatment restriction
cs_date_1              Additionally adjusted for treatment restriction
cs_date_2              Additionally adjusted for treatment restriction
cs_date_3              Additionally adjusted for treatment restriction
                                         term     estimate         se
Current_or_x_smokerYes Current_or_x_smokerYes  0.128324214 0.20163482
CCI                                       CCI -0.015365947 0.05558454
SAPS3                                   SAPS3  0.019598899 0.01018439
BMI                                       BMI -0.032443001 0.01917448
Age                                       Age  0.018403839 0.01072070
Woman0                                 Woman0  0.003267352 0.19474609
Treatment_restr               Treatment_restr  2.454609334 0.20731123
SjukhusHospital C2         SjukhusHospital C2  0.705254982 0.34449047
SjukhusHospital C1         SjukhusHospital C1  0.906922305 0.37000812
SjukhusHospital A1         SjukhusHospital A1  0.818978256 0.33670178
SjukhusHospital B1         SjukhusHospital B1  0.837131754 0.33003135
SjukhusHospital B3         SjukhusHospital B3  1.142466533 0.35213014
SjukhusHospital C3         SjukhusHospital C3  1.193493922 0.58060880
cs_date_1                           cs_date_1  0.311871732 0.42422696
cs_date_2                           cs_date_2 -3.051010481 1.72807974
cs_date_3                           cs_date_3 -0.412209778 0.67115868
                               HR        LCL       UCL            p n_mean
Current_or_x_smokerYes  1.13692155 0.765764937  1.687973 5.245034e-01    747
```

```
CCI                        0.98475151 0.883104590  1.098098 7.822080e-01    747
SAPS3                      1.01979222 0.999637556  1.040353 5.430379e-02    747
BMI                        0.96807763 0.932370535  1.005152 9.064808e-02    747
Age                        1.01857423 0.997394673  1.040204 8.604049e-02    747
Woman0                     1.00327270 0.684932500  1.469570 9.866141e-01    747
Treatment_restr           11.64188457 7.754546487 17.477937 2.418357e-32    747
SjukhusHospital C2         2.02436280 1.030509828  3.976716 4.063444e-02    747
SjukhusHospital C1         2.47668830 1.199262018  5.114800 1.424253e-02    747
SjukhusHospital A1         2.26818115 1.172388094  4.388176 1.500100e-02    747
SjukhusHospital B1         2.30973259 1.209576504  4.410523 1.119597e-02    747
SjukhusHospital B3         3.13449016 1.571910021  6.250376 1.176801e-03    747
SjukhusHospital C3         3.29858610 1.057069731 10.293238 3.982201e-02    747
cs_date_1                  1.36597947 0.594750608  3.137281 4.622463e-01    747
cs_date_2                  0.04731109 0.001599528  1.399375 7.747144e-02    747
cs_date_3                  0.66218535 0.177696047  2.467638 5.390988e-01    747
                         events_mean n_parameters  EPV
Current_or_x_smokerYes           148           16 9.25
CCI                              148           16 9.25
SAPS3                            148           16 9.25
BMI                              148           16 9.25
Age                              148           16 9.25
Woman0                           148           16 9.25
Treatment_restr                  148           16 9.25
SjukhusHospital C2               148           16 9.25
SjukhusHospital C1               148           16 9.25
SjukhusHospital A1               148           16 9.25
SjukhusHospital B1               148           16 9.25
SjukhusHospital B3               148           16 9.25
SjukhusHospital C3               148           16 9.25
cs_date_1                        148           16 9.25
cs_date_2                        148           16 9.25
cs_date_3                        148           16 9.25
```

```r
## Excluding transfers###

est_excl <- list()
vcov_excl <- list()

N_excl <- integer(m)
E_excl <- integer(m)

for (k in 1:m) {
  d_k <- complete(imp, k)

  ## Exclude patients with recorded treatment restrictions
  d_k <- d_k[!is.na(d_k$Treatment_restr) & d_k$Treatment_restr != 2, ]

  ## Drop unused factor levels after subsetting
  d_k$Sjukhus <- droplevels(d_k$Sjukhus)
  d_k$Sjukvardsregion <- droplevels(d_k$Sjukvardsregion)
  d_k$Current_or_x_smoker <- droplevels(d_k$Current_or_x_smoker)
  d_k$Woman <- droplevels(d_k$Woman)

  fit_k <- coxme::coxme(
```

```r
    Surv(Tid_censur_event, Ninety_day_mortality) ~
      Current_or_x_smoker + CCI + SAPS3 + BMI + Age + Woman +
      Sjukhus + (1 | Sjukvardsregion) +
      cs_date_1 + cs_date_2 + cs_date_3,
    data = d_k
  )

  b <- stats::coef(fit_k)
  V <- as.matrix(vcov(fit_k))

  est_excl[[k]] <- b
  vcov_excl[[k]] <- V

  N_excl[k] <- nrow(d_k)
  E_excl[k] <- sum(d_k$Ninety_day_mortality == 1, na.rm = TRUE)
}

comb_excl <- pool_rubin(est_excl, vcov_excl)

res_excl <- data.frame(
  model = "Excluding patients with treatment restrictions",
  term = names(comb_excl$estimates),
  estimate = as.numeric(comb_excl$estimates),
  se = sqrt(diag(comb_excl$variances))
)

res_excl$HR  <- exp(res_excl$estimate)
res_excl$LCL <- exp(res_excl$estimate - 1.96 * res_excl$se)
res_excl$UCL <- exp(res_excl$estimate + 1.96 * res_excl$se)
res_excl$p   <- 2 * pnorm(-abs(res_excl$estimate / res_excl$se))

res_excl$n_mean <- mean(N_excl)
res_excl$events_mean <- mean(E_excl)
res_excl$n_parameters <- length(comb_excl$estimates)
res_excl$EPV <- mean(E_excl) / length(comb_excl$estimates)

write.csv(
  res_excl,
  "coxme_excluding_treatment_restrictions.csv",
  row.names = FALSE
)

res_excl
```

```
                                                               model
Current_or_x_smokerYes Excluding patients with treatment restrictions
CCI                    Excluding patients with treatment restrictions
SAPS3                  Excluding patients with treatment restrictions
BMI                    Excluding patients with treatment restrictions
Age                    Excluding patients with treatment restrictions
Woman0                 Excluding patients with treatment restrictions
SjukhusHospital C2     Excluding patients with treatment restrictions
SjukhusHospital C1     Excluding patients with treatment restrictions
SjukhusHospital A1     Excluding patients with treatment restrictions
```

```
SjukhusHospital B1     Excluding patients with treatment restrictions
SjukhusHospital B3     Excluding patients with treatment restrictions
SjukhusHospital C3     Excluding patients with treatment restrictions
cs_date_1              Excluding patients with treatment restrictions
cs_date_2              Excluding patients with treatment restrictions
cs_date_3              Excluding patients with treatment restrictions
                                         term     estimate         se
Current_or_x_smokerYes Current_or_x_smokerYes -0.099658253 0.38506662
CCI                                       CCI  0.176720077 0.08693537
SAPS3                                   SAPS3  0.050938489 0.01706384
BMI                                       BMI -0.005492469 0.02990626
Age                                       Age  0.034327376 0.01690377
Woman0                                 Woman0  0.079457060 0.35401158
SjukhusHospital C2         SjukhusHospital C2  0.841191270 0.60483210
SjukhusHospital C1         SjukhusHospital C1  0.233389375 0.60103079
SjukhusHospital A1         SjukhusHospital A1  0.925696334 0.49128620
SjukhusHospital B1         SjukhusHospital B1  0.483117667 0.57888590
SjukhusHospital B3         SjukhusHospital B3  1.519429706 0.56660160
SjukhusHospital C3         SjukhusHospital C3  1.384665018 0.83173985
cs_date_1                           cs_date_1  0.643091604 0.88748515
cs_date_2                           cs_date_2 -4.958755731 3.16858409
cs_date_3                           cs_date_3  0.010324288 1.18550765
                                HR          LCL       UCL           p n_mean
Current_or_x_smokerYes 0.905146696 4.255432e-01  1.925282 0.795783524    604
CCI                    1.193297015 1.006347e+00  1.414977 0.042075233    604
SAPS3                  1.052258166 1.017647e+00  1.088046 0.002834194    604
BMI                    0.994522587 9.379030e-01  1.054560 0.854283233    604
Age                    1.034923360 1.001197e+00  1.069786 0.042280067    604
Woman0                 1.082699068 5.409623e-01  2.166948 0.822409013    604
SjukhusHospital C2     2.319128040 7.087307e-01  7.588714 0.164290707    604
SjukhusHospital C1     1.262873115 3.888230e-01  4.101734 0.697782810    604
SjukhusHospital A1     2.523624935 9.634597e-01  6.610222 0.059533833    604
SjukhusHospital B1     1.621120645 5.212640e-01  5.041653 0.403962913    604
SjukhusHospital B3     4.569618428 1.505147e+00 13.873338 0.007325909    604
SjukhusHospital C3     3.993487935 7.822780e-01 20.386545 0.095956377    604
cs_date_1              1.902353120 3.340785e-01 10.832626 0.468683725    604
cs_date_2              0.007021659 1.410219e-05  3.496174 0.117588709    604
cs_date_3              1.010377768 9.893713e-02 10.318303 0.993051512    604
                       events_mean n_parameters EPV
Current_or_x_smokerYes          45           15   3
CCI                             45           15   3
SAPS3                           45           15   3
BMI                             45           15   3
Age                             45           15   3
Woman0                          45           15   3
SjukhusHospital C2              45           15   3
SjukhusHospital C1              45           15   3
SjukhusHospital A1              45           15   3
SjukhusHospital B1              45           15   3
SjukhusHospital B3              45           15   3
SjukhusHospital C3              45           15   3
cs_date_1                       45           15   3
cs_date_2                       45           15   3
cs_date_3                       45           15   3
```
