# Supplementary material for: COVID‐19 Mortality in Swedish Intensive Care Units: A Multicenter Survival Analysis
Source: Acta Anaesthesiol Scand. 2026 Jun 14;70(6):e70279. doi: 10.1111/aas.70279 (PMC13265249; doi:10.1111/aas.70279)
Supplement: Supplementary file 5 — Data S5: STROBE statement—checklist of items that should be included in reports of observational studies. [file AAS-70-0-s009.docx]

STROBE Statement—checklist of items that should be included in reports of observational studies

|  | Item No. | Recommendation | Page  No. | Relevant text from manuscript |
| --- | --- | --- | --- | --- |
| **Title and abstract** | 1 | (*a*) Indicate the study’s design with a commonly used term in the title or the abstract | 1 | “..A Multicenter Survival Analysis…” |
|  |  | (*b*) Provide in the abstract an informative and balanced summary of what was done and what was found | 1 | Please see the abstract for a summary of what was done and what was found. |
| Introduction | | | |  |
| Background/rationale | 2 | Explain the scientific background and rationale for the investigation being reported | 3 | Please see the manuscript for the scientific background and rationale for the investigation being reported. |
| Objectives | 3 | State specific objectives, including any prespecified hypotheses | 3-4 | “We hypothesized that 90-day mortality among ICU-treated COVID-19 patients would vary between hospitals and that this variation would be related to patient-level case-mix. The aim of the present study was therefore to examine whether mortality differed according to the hospital of initial ICU admission using adjusted analyses accounting for patient-level factors, treating the hospital of admission as a contextual exposure reflecting difference in care environments.”  “Primary research question 1. Does 90-day mortality among COVID-19 ICU patients vary by the hospital of initial ICU admission, and does this variation remain after adjustment for case-mix? Secondary research questions 2. What inter-hospital differences in intensive care management of COVID-19 patients can be identified? 3. Do these differences align with the mortality variation observed between hospitals? “ |
| Methods | | | |  |
| Study design | 4 | Present key elements of study design early in the paper | 4 | “We performed a population-based multicentre retrospective cohort study including all ICUs from seven hospitals, within three healthcare counties in Sweden, covering a total population of one million inhabitants.” |
| Setting | 5 | Describe the setting, locations, and relevant dates, including periods of recruitment, exposure, follow-up, and data collection | 4 | “We performed a population-based multicentre retrospective cohort study including all ICUs from seven hospitals, within three healthcare counties in Sweden, covering a total population of one million inhabitants. County 1 includes three hospitals with ICUs at two of them (A1 and B1). County 2 comprises three hospitals equipped with ICUs (B2, C1, C2). County 3 includes three hospitals, two of which have ICUs (B3 and C3). Hospital A1 is a university hospital, B1-3 are county hospitals and C1-3 are local hospitals. All patients 18 years of age or older admitted to an ICU with acute hypoxaemic respiratory failure due to COVID-19 from 1 March 2020 to 31 July 2021 were included. The study inclusion period was chosen to capture the initial phase of the COVID-19 pandemic. At the time, ICU care pathways, treatment strategies and resource availability were still under development, providing an opportunity to study how early local management strategies and organisational responses potentially affected patient outcomes.” |
| Participants | 6 | (*a*) *Cohort study*—Give the eligibility criteria, and the sources and methods of selection of participants. Describe methods of follow-up  *Case-control study*—Give the eligibility criteria, and the sources and methods of case ascertainment and control selection. Give the rationale for the choice of cases and controls  *Cross-sectional study*—Give the eligibility criteria, and the sources and methods of selection of participants | 4-5 | “All patients 18 years of age or older admitted to an ICU with acute hypoxaemic respiratory failure due to COVID-19 from 1 March 2020 to 31 July 2021 were included.”  “COVID-19 is defined as rtPCR-confirmed infection with Severe respiratory syndrome coronavirus 2 (SARS-CoV-2).”  “Identification of patients was done by ICD codes of COVID-19 infection (both primary and secondary) on ICU admitted patients in SIR. All identified patients were manually screened and only those admitted due to COVID-19 hypoxemic respiratory failure were included.” |
|  |  | (*b*) *Cohort study*—For matched studies, give matching criteria and number of exposed and unexposed  *Case-control study*—For matched studies, give matching criteria and the number of controls per case |  | Not applicable |
| Variables | 7 | Clearly define all outcomes, exposures, predictors, potential confounders, and effect modifiers. Give diagnostic criteria, if applicable | 5 and 6 | “Hospital refers to the hospital and ICU of initial admission. 90-day mortality is defined as all-cause mortality within 90 days from ICU admission. Date of death was obtained through electronic medical journals and its linkage with the Swedish personal identity number. Acute respiratory distress syndrome (ARDS) was defined according to the Berlin definition (12), as adopted by the Swedish intensive care registry (SIR) (13) and reported as the most severe degree of ARDS developed. COVID-19 is defined as rtPCR-confirmed infection with Severe respiratory syndrome coronavirus 2 (SARS-CoV-2). Corticosteroid treatment was defined as any treatment started with dexamethasone, betamethasone, prednisolone, hydrocortisone or methylprednisolone for a duration of at least 48 hours. Comorbidity was defined as per the international classification of diseases and related health problems revision 10 (ICD-10) resulting in a Charlson comorbidity index (CCI) for participants, excluding age (14).  “ “To create a conceptual causal framework to guide confounder selection for the primary analysis of exposure, hospital at ICU admission and outcome (90-day mortality), a directed acyclic graph (DAG) was constructed (Figure 1). The DAG was constructed based on established principles of causal inference in epidemiology, including prior knowledge of relationships between baseline characteristics, care processes, and outcomes (16).  “ |
| Data sources/ measurement | 8* | For each variable of interest, give sources of data and details of methods of assessment (measurement). Describe comparability of assessment methods if there is more than one group | *5* | “Identification of patients was done by ICD codes of COVID-19 infection (both primary and secondary) on ICU admitted patients in SIR. All identified patients were manually screened and only those admitted due to COVID-19 hypoxemic respiratory failure were included. Clinical data were collected from medical records and from SIR. Variables included were age, sex, comorbidity, disease severity (Simplified Acute Physiology Score (SAPS3)), respiratory support (high-flow nasal oxygen (HFNO), non-invasive ventilation (NIV), invasive mechanical ventilation (IMV), tracheostomy), pharmacological treatment (corticosteroids, antibiotics, antivirals and anticoagulation), dialysis, complications (readmission, reintubation). Transfers between ICUs were registered as a dichotomous yes/no variable, and underlying reasons for transfers were not registered.” |
| Bias | 9 | Describe any efforts to address potential sources of bias | - | Several efforts were made, please See Methods, sections following “statistics”; see also Figure 1 for a directed acyclic graph. |
| Study size | 10 | Explain how the study size was arrived at | 4 | “All patients 18 years of age or older admitted to an ICU with acute hypoxaemic respiratory failure due to COVID-19 from 1 March 2020 to 31 July 2021 were included. The study inclusion period was chosen to capture the initial phase of the COVID-19 pandemic. At the time, ICU care pathways, treatment strategies and resource availability were still under development, providing an opportunity to study how early local management strategies and organisational responses potentially affected patient outcomes.” |

Continued on next page

| Quantitative variables | 11 | Explain how quantitative variables were handled in the analyses. If applicable, describe which groupings were chosen and why | 6 | “For all descriptive statistics, missing data were handled through listwise deletion. No analysis of missing data was performed for these analyses. Normally distributed continuous data are presented as mean (±SD), non-normally distributed as median (IQR) and categorical data as a percentage. A Kaplan-Meier curve was made using the survminer package in R.  “ |
| --- | --- | --- | --- | --- |
| Statistical methods | 12 | (*a*) Describe all statistical methods, including those used to control for confounding | 6-8 | “To create a conceptual causal framework to guide confounder selection for the primary analysis of exposure, hospital at ICU admission and outcome (90-day mortality), a directed acyclic graph (DAG) was constructed (Figure 1). The DAG was constructed based on established principles of causal inference in epidemiology, including prior knowledge of relationships between baseline characteristics, care processes, and outcomes (16).  The primary analysis used a Cox proportional hazards model with mixed effects, including a random intercept for healthcare county to account for unknown correlations within each county. To account for non-linear time-trends in case-mix and clinical practice during the study period, calendar time was modelled as a continuous variable using natural cubic splines with internal knots placed on July 1, 2020, and February 16 2021. Knot placement was decided based on local incidence peaks, corresponding to three pandemic waves. The model estimated the association between hospital of initial ICU admission (fixed effect) and 90-day mortality, adjusting for all covariates identified within the DAG. SAPS3 was entered as a linear variable within the model. Standardized 90-day mortality was estimated by model-based marginal standardization (g-computation) from fixed-effects Cox models refitted within each imputed dataset and pooled using Rubin’s rules. The resulting risks represent the predicted probability of death under hypothetical allocation of the cohort to each hospital, conditional on measured baseline covariates. Results from the survival analyses are presented as hazard ratios (95% CI) for the hospital of initial admission. For the healthcare county variable, healthcare county 2 was used as reference. For the hospital variable, Hospital B2 (the site with the lowest 90-day mortality) was used as reference. To assess potential overfitting, events per variable (EPV) was calculated for both primary and secondary exploratory models by dividing number of deaths at 90-days by the number of included regression coefficients.  “ |
|  |  | (*b*) Describe any methods used to examine subgroups and interactions | 7-8 | “Complete case analysis is included as a sensitivity analysis. Further, a complementary sensitivity model identical to the primary analysis but excluding all transferred patients was performed.  Secondary analysis  In addition to our primary model estimating the association between hospital of initial ICU admission and 90-day mortality, two secondary exploratory analyses including selected post-exposure, treatment-related variables were conducted. Due to limited sample size at most three variables were feasible to include: inter-hospital transfer, treatment restriction, time to intubation and time to corticosteroid initiation. These were identified a priori and informed by descriptive differences between included hospitals. The analysis was not a formal mediation analysis and cannot distinguish causality, given both time-dependent confounding and shared determinants of treatment timing and outcome. The purpose was to explore if inclusion of selected downstream factors attenuated the observed hospital-level association in our primary analysis. In a separate model, treatment restrictions as a binary variable were included for explorative purposes. As the timing of treatment restrictions were lacking in our dataset, it may have occurred both upstream and downstream of our exposure of interest (hospital). Accordingly, results from the secondary analyses should only be interpreted as hypothesis-generating. Further methodological details can be found in the supplementary methods.  “ |
|  |  | (*c*) Explain how missing data were addressed |  | “For variables included within the multivariable survival analysis, missingness was evaluated using graphical displays, correlation matrices of missing indicators and logistic regression models to assess whether data were missing completely at random (MCAR) or at random (MAR). In cases of substantial proportion of missing values, variables were imputed under the MAR assumption using multiple imputation by chained equations (mice package in R). For continuous variables, predictive mean matching was used. For binary variables, logistic regression was used. The imputation model included all variables in the final Cox model as predictors, together with calendar-time splines, hospitals and healthcare counties. Specifically, the imputation model for variable “CCI” included age, sex, smoking status, SAPS3, BMI, hospital (exposure), healthcare rgion, calendar-time splines and 90-day mortality. Thirty imputed datasets were generated and combined using Rubin’s rules. Complete case analysis is included as a sensitivity analysis. Further, a complementary sensitivity model identical to the primary analysis but excluding all transferred patients was performed.  “ |
|  |  | (*d*) *Cohort study*—If applicable, explain how loss to follow-up was addressed  *Case-control study*—If applicable, explain how matching of cases and controls was addressed  *Cross-sectional study*—If applicable, describe analytical methods taking account of sampling strategy |  | Not applicable |
|  |  | (*e*) Describe any sensitivity analyses | 7 | “For variables included within the multivariable survival analysis, missingness was evaluated using graphical displays, correlation matrices of missing indicators and logistic regression models to assess whether data were missing completely at random (MCAR) or at random (MAR). In cases of substantial proportion of missing values, variables were imputed under the MAR assumption using multiple imputation by chained equations (mice package in R). For continuous variables, predictive mean matching was used. For binary variables, logistic regression was used. The imputation model included all variables in the final Cox model as predictors, together with calendar-time splines, hospitals and healthcare counties. Specifically, the imputation model for variable “CCI” included age, sex, smoking status, SAPS3, BMI, hospital (exposure), healthcare rgion, calendar-time splines and 90-day mortality. Thirty imputed datasets were generated and combined using Rubin’s rules. Complete case analysis is included as a sensitivity analysis. Further, a complementary sensitivity model identical to the primary analysis but excluding all transferred patients was performed.  “ |
| Results | | | | |
| Participants | 13* | (a) Report numbers of individuals at each stage of study—eg numbers potentially eligible, examined for eligibility, confirmed eligible, included in the study, completing follow-up, and analysed | 9 | Please see figure 2 |
|  |  | (b) Give reasons for non-participation at each stage | 9 | Please see figure 2 |
|  |  | (c) Consider use of a flow diagram | 9 | Please see figure 2 |
| Descriptive data | 14* | (a) Give characteristics of study participants (eg demographic, clinical, social) and information on exposures and potential confounders | 8, 10 | “804 patients were identified, of which a total of 747 patients were included. A study flowchart is presented in Figure 2. Cohort characteristics and outcomes, stratified by hospital within each healthcare county, are shown in table 1.” + Table 1, page 10 |
|  |  | (b) Indicate number of participants with missing data for each variable of interest | 14 | Please see supplementary quarto documents for specific number of missing data for each variable of interest. Also, section “missing data and imputation” within main manuscript “Missingness was modest for most covariates (below 4%), except for smoking status and CCI (27 and 17 %, respectively). Logistic regressions displayed that missingness was associated with sex and hospital for smoking status, and with hospital and SAPS3 for CCI, supporting that data was missing at random (MAR). Due to this, missing values were imputed using chained equations (creating 30 datasets) with predictive mean matching for continuous variables and logistic regression for binary variables. Multiple imputation yielded stable convergence (see trace plots, supplementary file “Survival analysis”), and the mean fraction of missing information was below 9% for all variables.  “ |
|  |  | (c) *Cohort study*—Summarise follow-up time (eg, average and total amount) |  | Not applicable |
| Outcome data | 15* | *Cohort study*—Report numbers of outcome events or summary measures over time | *10,11* | *Please see figure 3 and table 1* |
|  |  | *Case-control study—*Report numbers in each exposure category, or summary measures of exposure |  | *Not applicable* |
|  |  | *Cross-sectional study—*Report numbers of outcome events or summary measures |  | *Not applicable* |
| Main results | 16 | (*a*) Give unadjusted estimates and, if applicable, confounder-adjusted estimates and their precision (eg, 95% confidence interval). Make clear which confounders were adjusted for and why they were included | - | Please see figure 1 for variable selection. Unadjusted estimates can be found in table 1 and figure 3, adjusted estimates within table 3. |
|  |  | (*b*) Report category boundaries when continuous variables were categorized |  | Not applicable |
|  |  | (*c*) If relevant, consider translating estimates of relative risk into absolute risk for a meaningful time period | 14 | Through marginal standardization of model-based predictions, estimated absolute mortality risk for each hospital is presented within table 4. |

Continued on next page

| Other analyses | 17 | Report other analyses done—eg analyses of subgroups and interactions, and sensitivity analyses | 13-15 | Reported under sections “Missing data and imputation  “Secondary analysis  “ and “Sensitivity analysis and robustness  “ |
| --- | --- | --- | --- | --- |
| Discussion | | | | |
| Key results | 18 | Summarise key results with reference to study objectives | 15 | “In this multicentre cohort study of critically ill COVID-19 patients, we present significant differences in 90-day mortality between the hospitals at which patients were initially admitted, irrespective of subsequent inter-hospital transfers. One hospital presented with significantly lower 90-day mortality than all other included units. The increased hazard persisted after adjustment for baseline confounders, calendar time and healthcare county. The results were robust across sensitivity analyses and indicate a contextual association related to hospital of initial ICU admission, possibly reflecting a combination of organisational characteristics, clinical routines, ICU strain, and admission thresholds that are not captured in the current dataset.  “ |
| Limitations | 19 | Discuss limitations of the study, taking into account sources of potential bias or imprecision. Discuss both direction and magnitude of any potential bias | 18 | Several limitations are discussed at length at p18, please see the manuscript. |
| Interpretation | 20 | Give a cautious overall interpretation of results considering objectives, limitations, multiplicity of analyses, results from similar studies, and other relevant evidence | 15-18 | Please see the entire discussion section, and the conclusion  “Among patients admitted to ICU due to COVID-19, we observed a difference in mortality related to the hospital of first-ICU of admission. This difference persisted after adjustment for calendar time, baseline confounders and healthcare county. Potential explanations are lacking within the current study. Future studies should focus on comprehensive evaluation of both organizational and contextual determinants of mortality.  “ |
| Generalisability | 21 | Discuss the generalisability (external validity) of the study results | 18 | “Lastly, our analyses specifically focus on the pre-Omicron phase of the pandemic. Although vaccination programs were gradually implemented and various SARS-CoV-2 variants circulated during the study period, we lacked sufficiently detailed or complete data on individual vaccination status and viral variant to explicitly account for these factors. And, as differences in referral patterns, ICU organization, case-mix, pandemic burden and resource availability may influence both mortality and inter-hospital variation, our results may not be directly generalizable to other Swedish or Scandinavian ICUs. “ |
| Other information | |  | | |
| Funding | 22 | Give the source of funding and the role of the funders for the present study and, if applicable, for the original study on which the present article is based | 19 | “The study was funded by Region Östergötland (ref 978201, 1005642) and the Medical Research Council of Southeast Sweden (ref FORSS-941384, -964312, -968674, -1013279). Clinical Studies Sweden/Swedish Research Council 2022.” |

*Give information separately for cases and controls in case-control studies and, if applicable, for exposed and unexposed groups in cohort and cross-sectional studies.

**Note:** An Explanation and Elaboration article discusses each checklist item and gives methodological background and published examples of transparent reporting. The STROBE checklist is best used in conjunction with this article (freely available on the Web sites of PLoS Medicine at http://www.plosmedicine.org/, Annals of Internal Medicine at http://www.annals.org/, and Epidemiology at http://www.epidem.com/). Information on the STROBE Initiative is available at www.strobe-statement.org.
