# Supplementary material for: COVID‐19 Mortality in Swedish Intensive Care Units: A Multicenter Survival Analysis
Source: Acta Anaesthesiol Scand. 2026 Jun 14;70(6):e70279. doi: 10.1111/aas.70279 (PMC13265249; doi:10.1111/aas.70279)
Supplement: Supplementary file 6 — Data S6: Supplementary methods. [file AAS-70-0-s008.docx]

**Supplementary Methods**

*Sensitivity analysis and model diagnostics*

For the multiple imputation, convergence and plausibility were checked with trace plots and density/strip plots across imputations, and by calculating the mean fraction of missing information. To assess possible bias introduced by multiple imputation and determine result robustness, a complete case analysis using an identical Cox proportional hazards model but including only individuals with non-missing data on all covariates was performed. Further, proportional hazards assumption was evaluated using Schoenfeld residuals, and multicollinearity was assessed using variance-inflation factors (VIF). Given the potential for unmeasured confounding (as one baseline confounder, ethnicity, was unavailable), we quantified robustness using E-values. For each hospital, an E-value for the point estimate was computed. The E-values represent the minimum strength of association (on a risk-ratio scale) that an unmeasured confounder would need to have with both hospital of initial admission and mortality, to fully explain the observed association. Further description of sensitivity analysis and model diagnostics is available in supplements and attached Quarto files.

*Secondary analysis*

In addition to the primary model estimating the total effect of hospital on mortality, we constructed a secondary, exploratory model to account for potential mediation through selected treatment-related factors. Missing data were handled identically to the main analysis using multiple imputation by chained equations (m = 30). Because some mediators varied over time, the dataset was expanded into a start–stop (time-dependent) structure using the tmerge function in R. The model included the same covariates as the main model (age, sex, SAPS3, CCI, BMI, smoking, calendar splines, and hospital) and, in addition, three treatment-related variables identified a priori and from descriptive analyses: inter-hospital transfer, days to intubation, and days to corticosteroid initiation. The joint effect of hospital was evaluated using a pooled Wald test for all hospital coefficients combined. The secondary analysis was considered exploratory, and results from this model should be interpreted as hypothesis-generating.

To explore treatment restrictions and their effect on our main results, another exploratory model was built including the same variables as the primary analysis with the addition of treatment restriction (yes/no). Results can be found within supplementary table 4.
