# Supplementary material for: COVID‐19 Mortality in Swedish Intensive Care Units: A Multicenter Survival Analysis
Source: Acta Anaesthesiol Scand. 2026 Jun 14;70(6):e70279. doi: 10.1111/aas.70279 (PMC13265249; doi:10.1111/aas.70279)
Supplement: Supplementary file 7 — Table S1: Complete case random‐effects Cox regression model (n = 401) analyzing inter‐hospital differences in 90‐day mortality. Random intercept for healthcare county. Initial hospital of ICU admission, pandemic wave and baseline covariates as fixed effects. [file AAS-70-0-s005.docx]

**Supplementary Table 1.** Complete case random-effects Cox regression model (n=401) analysing inter-hospital differences in 90-day mortality. Random intercept for healthcare county. Initial hospital of ICU admission, pandemic wave and baseline covariates as fixed effects.

| **Variable** | **HR (CI)** |
| --- | --- |
| Hospital C2 | 4.863 (0.98-24.13) |
| Hospital C1 | 5.347 (1.14-25.02) |
| Hospital A1 | 4.332 (0.99-19.94) |
| Hospital B1 | 7.15 (1.65-31.02) |
| Hospital B3 | 14.92 (3.31-67.21) |
| Hospital C3 | 17.99 (3.20-101.29) |

HR (Hazard ratio) >1 indicates higher mortality relative to reference hospital B2. Random intercept for healthcare county, estimated variance= 0.00897. Baseline covariates are CCI, SAPS3 upon ICU admission, age, sex, smoking status and BMI. For pandemic wave, three splines with two internal knots at Juli 1 2020 and February 16 2021 was used.
