# Supplementary material for: COVID‐19 Mortality in Swedish Intensive Care Units: A Multicenter Survival Analysis
Source: Acta Anaesthesiol Scand. 2026 Jun 14;70(6):e70279. doi: 10.1111/aas.70279 (PMC13265249; doi:10.1111/aas.70279)
Supplement: Supplementary file 9 — Table S3: Exploratory time‐dependent Cox regression including treatment‐related variables, baseline covariates, initial hospital of ICU admission and pandemic wave. Analyses performed on multiply imputed data (m = 30) and pooled using Rubin's rules. [file AAS-70-0-s001.docx]

**Supplementary Table 3.** Exploratory time-dependent Cox regression including treatment-related variables, baseline covariates, initial hospital of ICU admission and pandemic wave. Analyses performed on multiply imputed data (m=30) and pooled using Rubin’s rules.

|  | **HR (CI)** |
| --- | --- |
| Hospital C2 | 4,35 (2,20-8,59) |
| Hospital C1 | 2,73 (1,31-5,69) |
| Hospital A1 | 2,73 (1,43-5,22) |
| Hospital B1 | 4,04 (2,19-7,47) |
| Hospital B3 | 6,39 (3,28-12,45) |
| Hospital C3 | 6,60 (2,08-20,89) |

HR (Hazard ratio) >1 indicates higher mortality relative to reference hospital B2. Baseline covariates are CCI, SAPS3 upon ICU admission, age, sex, smoking status and BMI. For pandemic wave, three splines with two internal knots at Juli 1 2020 and February 16 2021 was used. The three included treatment-related mediators were: Inter-hospital transfer, days to intubation, and days to corticosteroid initiation. Events per variable 8.2.
