# Supplementary material for: COVID‐19 Mortality in Swedish Intensive Care Units: A Multicenter Survival Analysis
Source: Acta Anaesthesiol Scand. 2026 Jun 14;70(6):e70279. doi: 10.1111/aas.70279 (PMC13265249; doi:10.1111/aas.70279)
Supplement: Supplementary file 10 — Table S4: Exploratory time‐dependent Cox regression including treatment restriction in addition to baseline covariates, initial hospital of ICU admission and pandemic wave. Analyses performed on multiply imputed data (m = 30) and pooled using Rubin's rules. [file AAS-70-0-s003.docx]

**Supplementary Table 4.** Exploratory time-dependent Cox regression including treatment restriction in addition to baseline covariates, initial hospital of ICU admission and pandemic wave. Analyses performed on multiply imputed data (m=30) and pooled using Rubin’s rules.

|  | **HR (CI)** |
| --- | --- |
| Hospital C2 | 2,02 (1,03-3,97) |
| Hospital C1 | 2,48 (1,20-5,11) |
| Hospital A1 | 2,27 (1,17-4,39) |
| Hospital B1 | 2,31 (1,21-4,41) |
| Hospital B3 | 3,13 (1,57-6,25) |
| Hospital C3 | 3,30 (1,06-10,29) |

HR (Hazard ratio) >1 indicates higher mortality relative to reference hospital B2. Baseline covariates are CCI, SAPS3 upon ICU admission, age, sex, smoking status and BMI. For pandemic wave, three splines with two internal knots at Juli 1 2020 and February 16 2021 was used. In addition, treatment restriction during before or during ICU care is included as an exploratory variable.
